# BCL11B suppresses tumor progression and stem cell traits in hepatocellular carcinoma by restoring p53 signaling activity

**DOI:** 10.1038/s41419-020-03115-3

**Published:** 2020-10-22

**Authors:** Wen-Jing Yang, Yun-Fan Sun, An-Li Jin, Li-Hua Lv, Jie Zhu, Bei-Li Wang, Yan Zhou, Chun-Yan Zhang, Hao Wang, Bo Hu, Peng-Xiang Wang, Liu Te, Bai-Shen Pan, Jian Zhou, Jia Fan, Xin-Rong Yang, Wei Guo

**Affiliations:** 1 Department of Laboratory Medicine, Zhongshan Hospital, Fudan University, Shanghai, China; 2 Department of Liver Surgery & Transplantation, Liver Cancer Institute, Zhongshan Hospital, Fudan University; Key Laboratory of Carcinogenesis and Cancer Invasion, Ministry of Education, 200032 Shanghai, P. R. China; 3 Shanghai Geriatric Institute of Chinese Medicine, Shanghai University of Traditional Chinese Medicine, 200031 Shanghai, China

**Keywords:** Cancer stem cells, Liver cancer, Cell growth

## Abstract

Accumulating evidence indicates that hepatocellular carcinoma (HCC) tumorigenesis, recurrence, metastasis, and therapeutic resistance are strongly associated with liver cancer stem cells (CSCs), a rare subpopulation of highly tumorigenic cells with self-renewal capacity and differentiation potential. Previous studies identified B cell leukemia/lymphoma-11b (BCL11B) as a novel tumor suppressor with impressive capacity to restrain CSC traits. However, the implications of BCL11B in HCC remain unclear. In this study, we found that low BCL11B expression was an independent indicator for shorter overall survival (OS) and time to recurrence (TTR) for HCC patients with surgical resection. In vitro and in vivo experiments confirmed BCL11B as a tumor suppressor in HCC with inhibitory effects on proliferation, cell cycle progression, apoptosis, and mobility. Furthermore, BCL11B could suppress CSC traits, as evidenced by dramatically decreased tumor spheroid formation, self-renewal potential and drug resistance. A Cignal Finder Array and dual-luciferase activity reporter assays revealed that BCL11B could activate the transcription of P73 via an E2F1-dependent manner. Thus, we concluded that BCL11B is a strong suppressor of retaining CSC traits in HCC. Ectopic expression of BCL11B might be a promising strategy for anti-HCC treatment with the potential to cure HBV-related HCC regardless of P53 mutation status.

## Introduction

Hepatocellular carcinoma (HCC) is the fifth most common malignancy worldwide^1^. Surgical resection remains the most effective approaches for curing HCC^2^, while its long-term efficacy is limited by a high frequency (~50–70%) of metastasis/recurrence within 5 years after operation^3,4^. Such predicament can be mainly attributed to a poor understanding of liver carcinogenesis and HCC molecular pathogenesis^5^. Therefore, there is an urgent need to clarify the molecular mechanisms of HCC progression so that new therapies can be developed.

Cancer stem cells (CSCs) are a rare subclass of highly tumorigenic cells with self-renewal capacity and differentiation potential^6,7^. HCC tumorigenesis, progression and drug resistance are strongly associated with liver CSCs^8^. Therefore, specific eradication of CSCs represents an appealing strategy for HCC treatment^9–12^. Dysfunction of tumor suppressor has been identified to play essential roles in restraining the self-renewal capacity of CSCs^13,14^, and P53 exerts strong power in hindering the stemness of HCC cells^15^. Previously, we found that 58% of Chinese patients with HBV-related HCC harbored P53 mutations^16^, indicating that abnormal P53 function is common in HCC. P53 dysfunction was shown to greatly contribute to the maintenance of CSC traits in HCC^17,18^. Those results suggest restoration of P53 signaling might be a promising approach to eliminate CSCs in HCC. However, currently, no effective therapeutic regimens are available to reactivate P53 signaling in HCC.

B cell leukemia/lymphoma-11b (BCL11B) is a C2H2-type transcription factor that has been identified as a critical regulator for T-cell acute lymphoblastic leukemia (T-ALL)^19,20^. BCL11B was recently shown to drive human mammary stem cell self-renewal by inhibiting basal differentiation^21^. In addition, BCL11B could inhibited LGR5 expression and downregulated the activity of the β-catenin pathway, thereby attenuating cell regeneration and impairing tumor development in colorectal cancer^22^. Although BCL11B was preferentially decreased and low BCL11B expression indicated poorer prognosis in LIHC (liver hepatocellular carcinoma) cohort according to TCGA database, its exact function in HCC remained elusive. Since HCC is characterized by its impressively stem cell traits and highly metastatic potentials, we focus our attention on exploring the biological functions of BCL11B in HCC progression. Here, we reported that the low BCL11B expression was an independent indicator for poor prognosis in HCC after surgical resection. Furthermore, BCL11B could activate the transcription of P73, a homologous protein of P53, resulting in suppression of its target genes such as P21 and CDK2, thereby exerting substantial inhibitory effects on the proliferation, migration, and stemness potential in HCC. Thus, targeting BCL11B might provide novel insights into HCC progression and metastasis, with potentially major therapeutic implications.

## Results

### BCLL11B downregulation is common in HCC and indicates poor prognosis

**Fig. 1:**
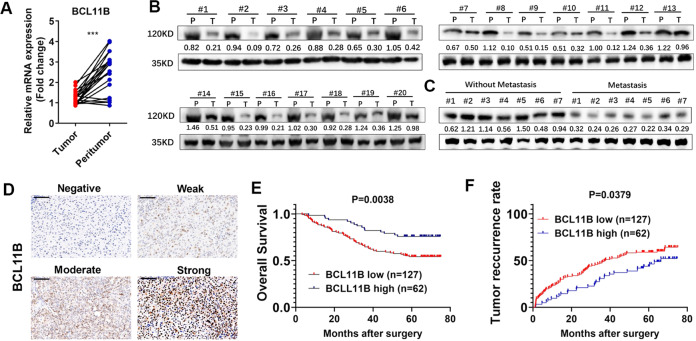
Downregulation of BCL11B in HCC tissues. **a** RT-PCR detection of BCL11B mRNA expression in 20 HCC tissues and matched non-tumor liver specimens. **b** The protein expression of BCL11B in 20 pairs of HCC tumor tissues (T) and corresponding peritumoral tissues (P). **c** Comparison of the relative expression levels of BCL11B protein between metastatic and non-metastatic HCC tissues. **d** Representative immunohistochemistry staining on BCL11B in 189 HCC patients. **e**, **f** Kaplan–Meier analysis of the overall survival (OS) (**e**) and the time-to-recurrence (TTR) (**f**) of HCC patients in BCL11B low group and BCL11B high group; Scale bar: 100 μm.

We first explore the expression pattern of BCL11B in TCGA database, and found that BCL11B expression was preferentially decreased in LIHC cohort (Supplementary Fig. 1a), and patients with low BCL11B expression had a significantly poorer prognosis (*P* < 0.05, Supplementary Fig. 1b). Next, BCL11B expression in 20 HCC and corresponding adjacent non-cancerous tissues were detected. Results showed that BCL11B was significantly downregulated in cancerous tissues (Fig. 1a, b). Moreover, primary tumor tissues of patients who suffered metastasis exhibited lower BCL11B protein levels (Fig. 1c). Next, prognostic value of BCL11B was determined based on TMA containing 189 patients. Representative images were shown in Fig. 1d. Patients were stratified into two groups according to their BCL11B expression level. Low BCL11B expression was significantly associated with advanced Edmondson stage (*P* = 0.012) and Barcelona Clinic Liver Cancer (BCLC) stage (*P* = 0.031; Supplementary Table 1). Kaplan–Meier analysis indicated patients with low BCL11B expression had significantly shorter OS and TTR (both *P* < 0.050, Fig. 1e, f). Univariate (Supplementary Table 2) and multivariate (Table 1) Cox proportional regression analyses revealed that BCL11B expression was an independent prognostic factor for OS (hazard ratio [HR] = 0.56, 95% confidence interval [CI] = 0.36–0.87, *P* = 0.009) and TTR (HR = 0.36, 95% CI = 0.20–0.65, *P* = 0.001).

**Table 1:** Multivariate cox proportional regression analysis of factors associated with recurrence and overall survival.

Variables	Recurrence		Overall survival	
	HR (95% CI)	P	HR (95% CI)	P
ALT (>40 U/L versus ≤40 U/L)	1.27 (0.82–1.97)	0.289	1.17 (0.68–2.03)	0.565
AST (>40 U/L versus ≤40 U/L)	1.30 (0.82–2.04)	0.265	1.24 (0.71–2.17)	0.456
AFP (>400 ng/ml versus ≤400 ng/ml)	1.98 (1.30–3.03)	**0.002**	1.66 (0.99–2.79)	0.053
No. of tumors (multi versus single)	1.42 (0.80–2.53)	0.236	1.41 (0.71–2.81)	0.329
Tumor size (>5 cm versus ≤5 cm)	1.45 (0.92–2.28)	0.113	0.89 (0.51–1.55)	0.682
Vascular invasion (yes versus no)	1.40 (0.90–2.18)	0.133	1.73 (1.02–2.91)	**0.041**
Edmondson stage (III–IV versus I–II)	1.62 (1.09–2.41)	**0.017**	1.38 (0.86–2.23)	0.186
BCL11B (Low versus high)	0.56 (0.36–0.87)	**0.009**	0.36 (0.20–0.65)	**0.001**

*ALT* alanine aminotransferase, *AST* aspartate transaminase, *AFP* α-fetoprotein, *BCLC* Barcelona Clinic Liver Cancer, *HR* hazard ratio.

The bold values were considered statistically significant (*P* < 0.05).

### BCLL11B inhibits proliferation, induces G0/G1 arrest, and attenuates cell mobility in HCC

**Fig. 2:**
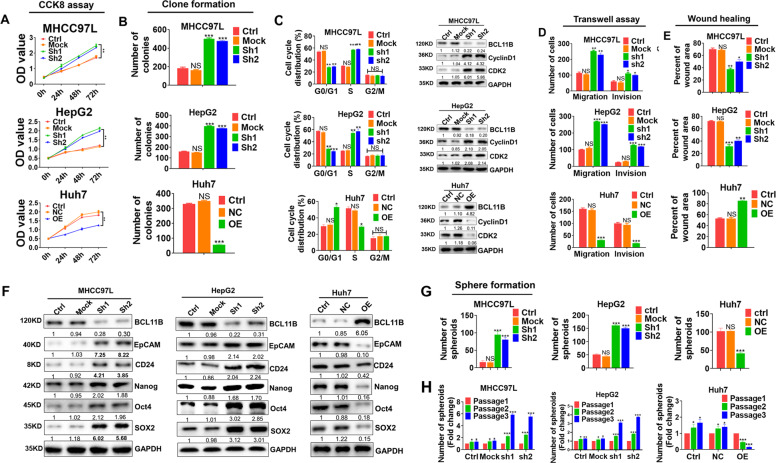
BCL11B inhibits proliferation, induces G0/G1 arrest and attenuates mobility and cell self-renewal capacities in HCC cells. **a** CCK-8 assay analysis of cell growth in BCL11B-knockdown MHCC97L and HepG2 cells, as well as BCL11B-overexpressing Huh7 cells. **b** Cell proliferation was detected by clone formation assay in indicated HCC cells to observe the effects of BCL11B expression on cell proliferation potentials. **c** Analysis of cell cycle in BCL11B-knockdown MHCC97L and HepG2 cells, as well as BCL11B-overexpressing Huh7 cells by flow cytometry (left), and western blot were conducted to evaluate expressions of cell cycle-related molecules (right). **d** Cell migration and invasion were analyzed by transwell assay in HCC cells according to indicated BCL11B expression manipulations. **e** Wound healing assays were conducted in indicated HCC cells to evaluate the effects of BCL11B expression manipulations on cell migration. **f** Dynamic changes of protein expression of CSC related markers due to BCL11B expression manipulations in indicated HCC cells. **g** Sphere-forming assays were conducted to evaluate the effects of BCL11B on CSC traits in HCC cells. **h** Self-renewal ability was evaluated by sphere-forming assays in three serial passages in MHCC97L- and HepG2-shBCL11B cells, as well as Huh7 BCL11B-OE cells. Corresponding parental cells were set as controls. Error bars represent the standard deviation (SD) from at least three independent experiments; *P < 0.05; **P < 0.01; ***P < 0.001.

BCL11B expressions in one normal liver cell line (L02) and seven HCC cell lines were determined. We found that BCL11B expression was dramatically downregulated in the HCC cell lines compared with that in the normal liver cell line (Supplementary Fig. 1c). We then transfected two HCC cell lines, HepG2 (P53 wild-type) and MHCC97L, which had relatively high BCL11B expression with two distinct short-hairpin RNAs targeting BCL11B (sh1 and sh2), in order to knock down BCL11B expression. Contrarily, we induced ectopic expression of BCL11B in Huh7 cells (P53 mutated). Empty lentiviral vehicle (Mock) was transfected as a control. BCL11B knockdown and overexpression efficiencies were confirmed by RT-PCR and Western blot assays (Supplementary Fig. 1d). BCL11B knockdown significantly enhanced the proliferation of MHCC97L and HepG2 cells (Fig. 2a, b and Supplementary Fig. 1e). Conversely, enforced overexpression of BCL11B inhibited the proliferation of Huh7 cells. Flow cytometry analysis indicated that BCL11B knockdown resulted in a substantial accumulation of MHCC97L cells and HepG2 cells in S phase, accompanied by a substantial decrease in the numbers of cells in G0/G1 phase. BCL11B overexpression achieved almost the opposite effects (Fig. 2c and Supplementary Fig. 1f). Furthermore, cell cycle-related protein expressions were evaluated by western blot. BCL11B knockdown significantly increased cyclin D1 and CDK2 expression, whereas BCL11B overexpression decreased cyclin D1 and CDK2 expression (Fig. 2c).

Next, transwell assays indicated that BCL11B knockdown markedly increased the number of migrating and invading MHCC97L and HepG2 cells, whereas BCL11B overexpression achieved the opposite effects in Huh7 cells (Fig. 2d and Supplementary Fig. 1g). Likewise, wound-healing assays revealed that BCL11B has an inhibitory effect on cell migration (*P* < 0.05; Fig. 2e and Supplementary Fig. 1h).

### BCL11B restrains the self-renewal potential of HCC cells

We next determined the expression levels of stemness-associated makers in BCL11B-modulated cells. BCL11B knockdown increased the expression of liver CSC-related markers (EpCAM and CD24) as well as that of a cluster of pluripotent stem cell markers (Nanog, OCT4, and SOX2). Conversely, BCL11B overexpression achieved the opposite effects (Fig. 2f and Supplementary Fig. 2a, b). Moreover, BCL11B knockdown significantly enhanced the number of spheroids formed in both MHCC97L and HepG2 cells (all *P* < 0.05; Fig. 2g and Supplementary Fig. 2c). Conversely, BCL11B overexpression significantly reduced the number of spheroids formed by Huh7 cells (both *P* < 0.001). Moreover, BCL11B-silenced MHCC97L cells exerted greater self-renewal ability over three serial passages compared with control cells, as evidenced by an increasing number of spheres during passaging (*P* < 0.001). We observed similar results when BCL11B was silenced in HepG2 cells (*P* < 0.001). By contrast, cells overexpressing BCL11B lost the ability of self-renewal, as evidenced by a decreasing number of spheres during serial passages (*P* < 0.001; Fig. 2h). Interestingly, silencing BCL11B expression in a normal hepatocyte-derived cell line (L02) also resulted in promoted effects on cell proliferation, mobility, and stemness (Supplementary Fig. 3a–e).

### BCL11B enhances the chemosensitivity of HCC cells

**Fig. 3:**
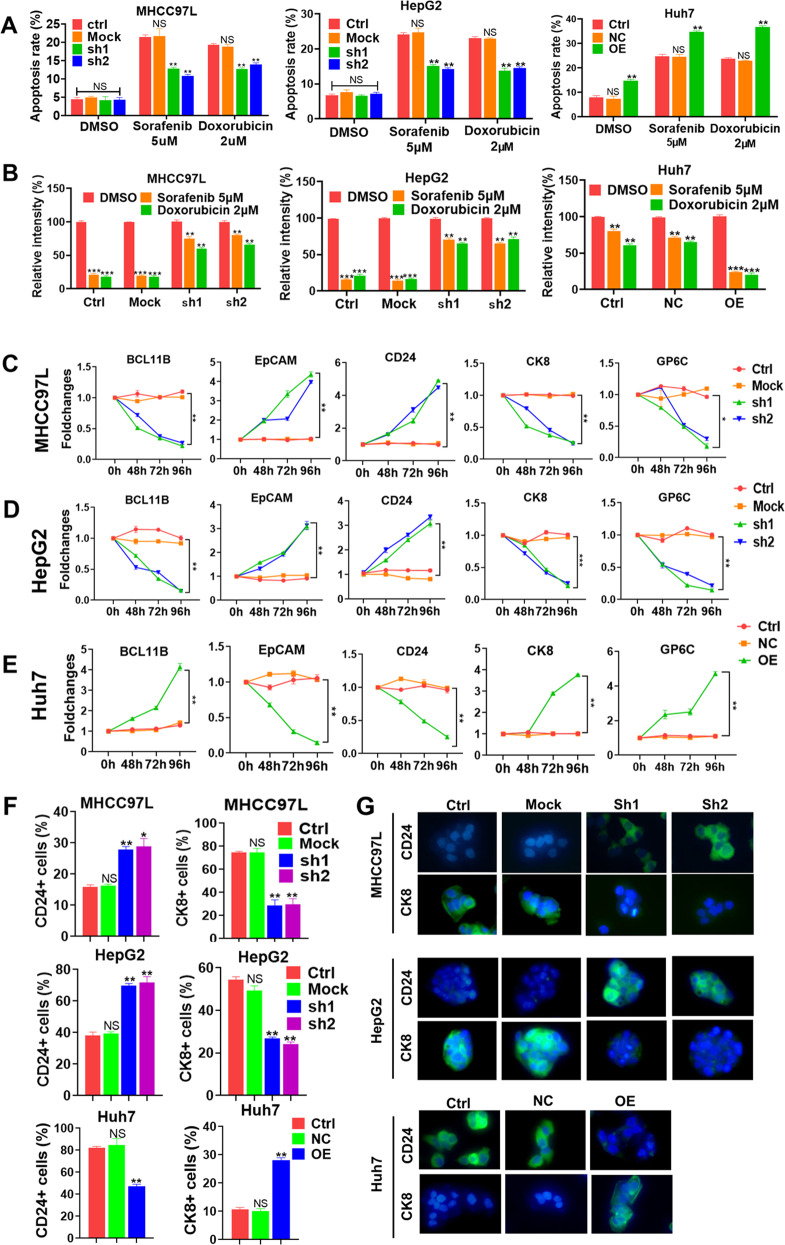
Silencing BCL11B enhanced chemosensitivity and induced cell differentiation in HCC. **a** BCL11B silencing resulted in lower percentages of apoptosis rate under 5 μM sorafenib or 2 μM doxorubicin treatment, while BCL11B overexpression led to higher percentages of apoptosis rate under 5 μM sorafenib or 2 μM doxorubicin treatment, compared with corresponding control cells, respectively. **b** Clone formation assays showed that BCL11B silencing resulted in higher numbers of clone under 5 μM sorafenib or 2 μM doxorubicin treatment, while BCL11B overexpression decreased number of Huh7 clone under 5 μM sorafenib or 2 μM doxorubicin treatment. **c**–**e** Dynamic changes of the protein expression levels of CSC markers (EpCAM and CD24) and mature hepatocyte markers (CK8 and GP6C) due to BCL11B expression manipulations in MHCC97L cells (**c**), HepG2 (**d**), and Huh7 cells (**e**). **f** Alterations of the percentage of CD24^+^ and CK8^+^ cells in indicated HCC cells due to BCL11B expression manipulations were evaluated by flow cytometry. **g** Immunofluorescence analysis of CD24 and CK8 expression in BCL11B-knockdown MHCC97L cells and HepG2 cells, as well as BCL11B-overexpressing Huh7 cells, with their corresponding parental cells as controls, respectively. Scale bar: 10 μm. Means ± SD from three independent experiments are presented; *P < 0.05; **P < 0.01; ***P < 0.001.

Drug resistance is a hallmark of CSCs^23^. Therefore, we investigated whether BCL11B expression increases the sensitivity of HCCs to the chemotherapeutic agents (sorafenib and doxorubicin). Compared with control cells, BCL11B-silenced MHCC97L cells exhibited lower apoptosis rates after treatment with 5 μM sorafenib or 2 μM doxorubicin. Similarly, BCL11B knockdown reduced the apoptosis rates of HepG2 cells after treatment with sorafenib or doxorubicin. Contrarily, BCL11B overexpression sensitized Huh7 cells towards chemotherapeutic treatment, as evidenced by increases in apoptosis rates from ~20% to almost 40% (Fig. 3a and Supplementary Fig. 4a). Moreover, MHCC97L and HepG2 cells with BCL11B knockdown had higher survival rates than wild-type or Mock-transfected parental cells after treatment with sorafenib or doxorubicin according to colony-formation assays (all *P* < 0.01). Conversely, Huh7 cells with BCL11B overexpression exhibited lower survival rates than parental or Mock-transfected cells after sorafenib or doxorubicin treatment (all *P* < 0.01; Fig. 3b and Supplementary Fig. 4b). Together, our results revealed that BCL11B expression greatly sensitizes HCC cells to congenital targeting or chemotherapy.

### BCL11B induces differentiation in HCC cells

It was reported that BCL11B could induce cell differentiation^21^. We then examined whether BCL11B has the same regulatory function in liver CSCs. Dynamic changes of several stemness-associated genes (EpCAM and CD24) and liver maturation-associated makers (CK8 and G6PC) after modulation of BCL11B expression were detected by WB assays. Results showed that BCL11B knockdown in MHCC97L and HepG2 cells increased the EpCAM and CD24 expressions in a time-dependent manner and decreased CK8 and GP6C expressions in a similar manner (all *P* < 0.01; Fig. 3c, d). By contrast, BCL11B overexpression in Huh7 cells had opposite effects on each of the four markers (all *P* < 0.01; Fig. 3e and Supplementary Fig. 5a).

To further confirm that BCL11B induces differentiation in liver CSCs, we performed flow cytometry to detect alterations in the percentages of CD24^+^ and CK8^+^ fractions after modulation of BCL11B expression. We found that BCL11B knockdown in MHCC97L cells and HepG2 cells greatly increased the percentages of CD24^+^ cells (all *P* < 0.01; Fig. 3f), whereas it dramatically reduced the percentages of CK8^+^ cells (all *P* < 0.01). Conversely, BCL11B overexpression in Huh7 cells greatly decreased the numbers of CD24^+^ cells and increased the numbers of CK8^+^ cells (*P* < 0.01; Supplementary Fig. 5b).

In agreement with the flow cytometry results, immunofluorescence assays also revealed that BCL11B induced differentiation in HCC cells. BCL11B knockdown in MHCC97L and HepG2 cells resulted in enhanced expression of CD24 and reduced expression of CK8, whereas BCL11B overexpression in Huh7 cells produced the opposite effects (Fig. 3g).

### BCL11B suppressed tumorigenesis and cancer cell self-renewal in vivo

**Fig. 4:**
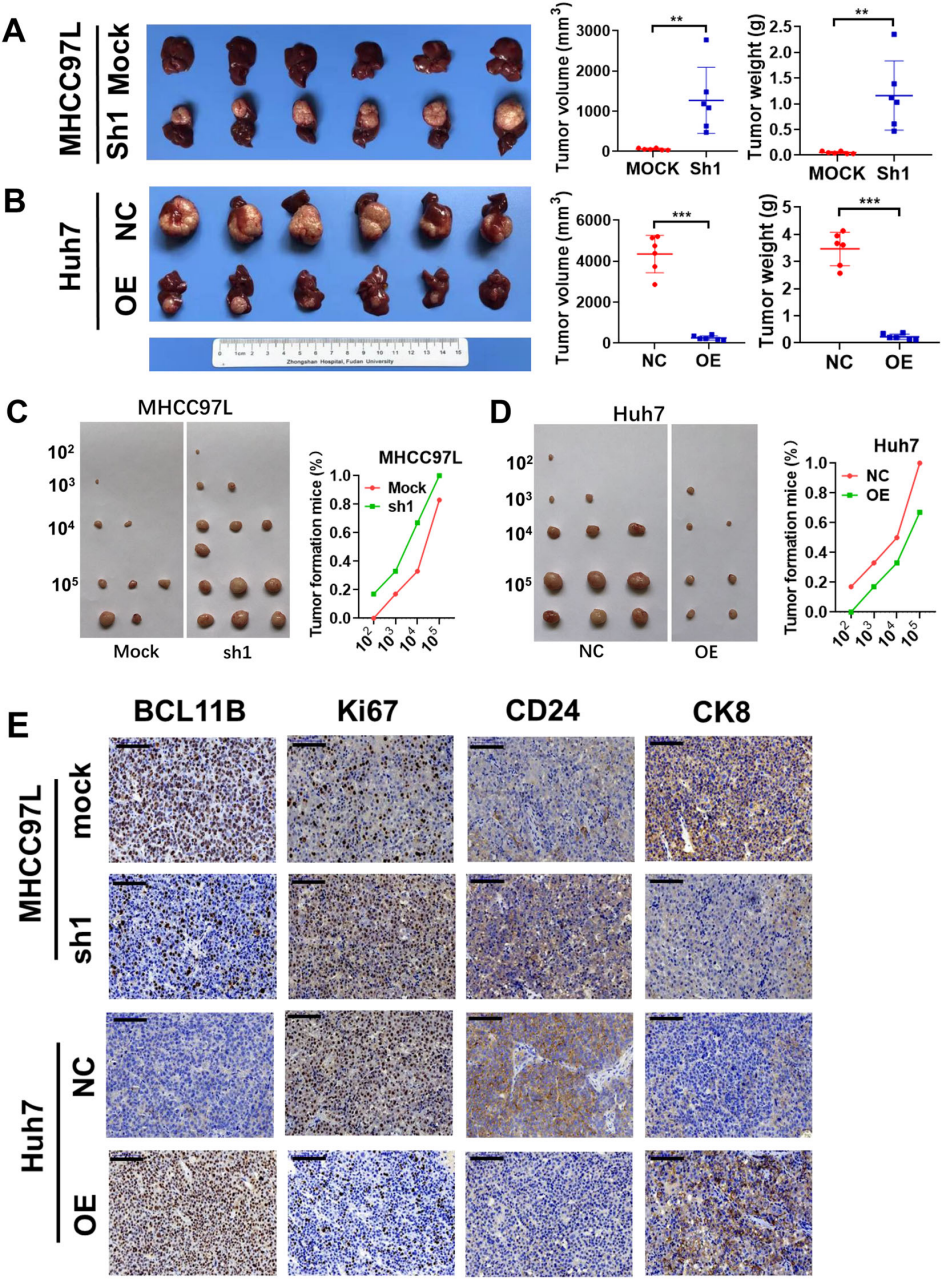
BCL11B inhibits cell proliferation and cell self-renewal capacities in vivo. **a** Knockdown of BCL11B in MHCC97L cells markedly promoted tumor growth in vivo according to orthotopic xenograft models. **b** Overexpression of BCL11B in Huh7 cells greatly inhibited tumor growth according to orthotopic xenograft models. **c** Nude mice injected with serially diluted BCL11B-knockdown MHCC97L cells (10^2^, 10^3^, 10^4^, and 10^5^) as well as corresponding parental cells to evaluate the effects of BCL11B expression on tumor initiation ability. **d** Nude mice injected with serially diluted BCL11B-overexpressing Huh7 cells (10^2^, 10^3^, 10^4^, and 10^5^) as well as corresponding parental cells to evaluate the effects of BCL11B expression on tumor initiation ability. **e** Expression states of cell proliferation (Ki67), cell stemness (CD24) and mature hepatocyte (CK8) markers were evaluated by IHC staining; *P < 0.05; **P < 0.01; ***P < 0.001.

In vivo experiments were performed to confirm the role of BCL11B in HCC. Huh7 cells stably overexpressing BCL11B, MHCC97L cells with stable BCL11B silencing, and their corresponding control cells were transplanted orthotopically into the livers of the nude mice. After 6 weeks, compared with those in the corresponding controls, the volumes and weights were significantly increased in the BCL11B-silenced MHCC97L tumors (*P* < 0.001; Fig. 4a) and decreased in the BCL11B-overexpressing Huh7 tumors (*P* < 0.001; Fig. 4b). To confirm that BCL11B restrains the traits of liver CSCs, we injected subcutaneously nude mice with serially diluted (10^2^, 10^3^, 10^4^, or 10^5^ cells) BCL11B-knockdown MHCC97L cells or BCL11B-overexpressing Huh7 cells. The BCL11B-silenced MHCC97L cells were able to generate tumors even when only 10^2^ cells were injected, and exhibited higher rates of tumor formation than control cells at all four dilution orders (Fig. 4c). Meanwhile, the BCL11B-overexpressing Huh7 cells failed to generate any tumors at the highest dilution, and they formed fewer tumors than control cells at all four dilutions (Fig. 4d). BCL11B, Ki67, CD24, and CK8 staining of the xenograft tumors were shown in Fig. 4e. Tumors with high BCL11B expression presented low CD24 and Ki67 expression and high CK8 expression. Tumors with low BCL11B expression exhibited the opposite expression patterns of CD24, Ki67, and CK8.

### BCL11B activated the transcription of P73, but not P53 in HCC

**Fig. 5:**
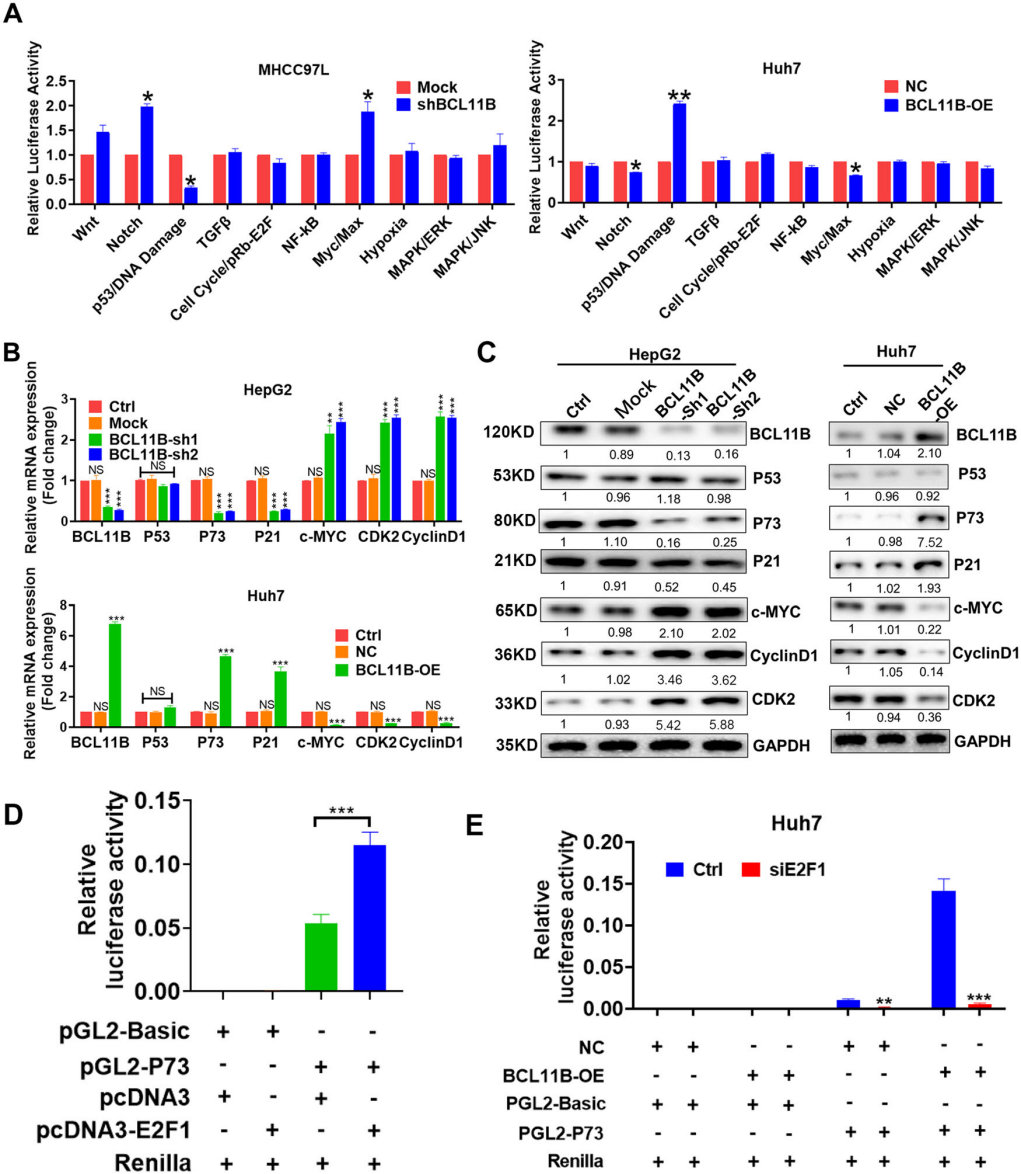
BCL11B activated P73 signaling in HCC. **a** Cignal Finder Center 10-Pathway Reporter Array results demonstrated signaling changes in the BCL11B-knockdown MHCC97L cells and BCL11B-overexpressing Huh7 cells. **b** mRNA expressions of P53 signaling-related molecules was detected in indicated HCC cells. **c** Protein expressions of P53 signaling-related molecules was detected in indicated HCC cells. **d** HEK293T cells were co-transfected with indicated plasmids to investigate promotor activity of P73. **e** Huh7 cells were co-transfected with indicated plasmids to investigate promotor activity of P73. Means ± SD from three independent experiments are presented; *P < 0.05; **P < 0.01; ***P < 0.001.

Cignal Finder Center 10-Pathway Reporter Array was used to explore the signaling pathways affected by BCL11B expression modulation. The P53 signaling pathway exhibited the greatest alterations (*P* < 0.05) in response to manipulations of BCL11B expression (Fig. 5a). Therefore, we hypothesized that BCL11B restrains CSC traits in HCC by affecting the P53 signaling pathway. To test that, we examined the effects of BCL11B on the P53 pathway in HepG2 cells (wild-type P53) and Huh7 cells (mutant P53). Surprisingly, although BCL11B expression had a strong influence on the expression of P53 target genes such as P21, c-MYC, and Cyclin D1, it shed no effect on P53 expression in both two cell lines (Fig. 5b, c). Those results indicated that BCL11B might suppress HCC regardless of the P53 mutation state, suggesting the BCL11B-dependent regulatory process might be P53-independent.

P73 exerted a compensatory effect when P53 function is compromised^24,25^, and its transcription process was P53-independent. Therefore, we further investigated whether BCL11B shed effects on P73 expression. Interestingly, we found that manipulation of BCL11B expression significantly altered P73 expression in HCC cells (Fig. 5b, c). Previously, E2F1 was reported as key regulator for P73 expression^26,27^, to further explore the potential regulatory mechanism of BCL11B on P73 transcription, we first co-transfected HEK293T cells with E2F1 and P73 luciferase reporter plasmids. Results confirmed E2F1 greatly increased P73 promoter activity (Fig. 5d), confirming its regulatory role. Notably, further experiments indicated E2F1 knockdown successfully abolished the enhancing effects of BCL11B overexpression on P73 signaling in BCL11B-overexpressing Huh7 cells (Fig. 5e). Taken together, the results indicated that BCL11B promotion of P73 transcription requires in the presence of E2F1.

### Knockdown of P73 rescued BCL11B-induced inhibitory effects on malignancy potentials in HCC cells

**Fig. 6:**
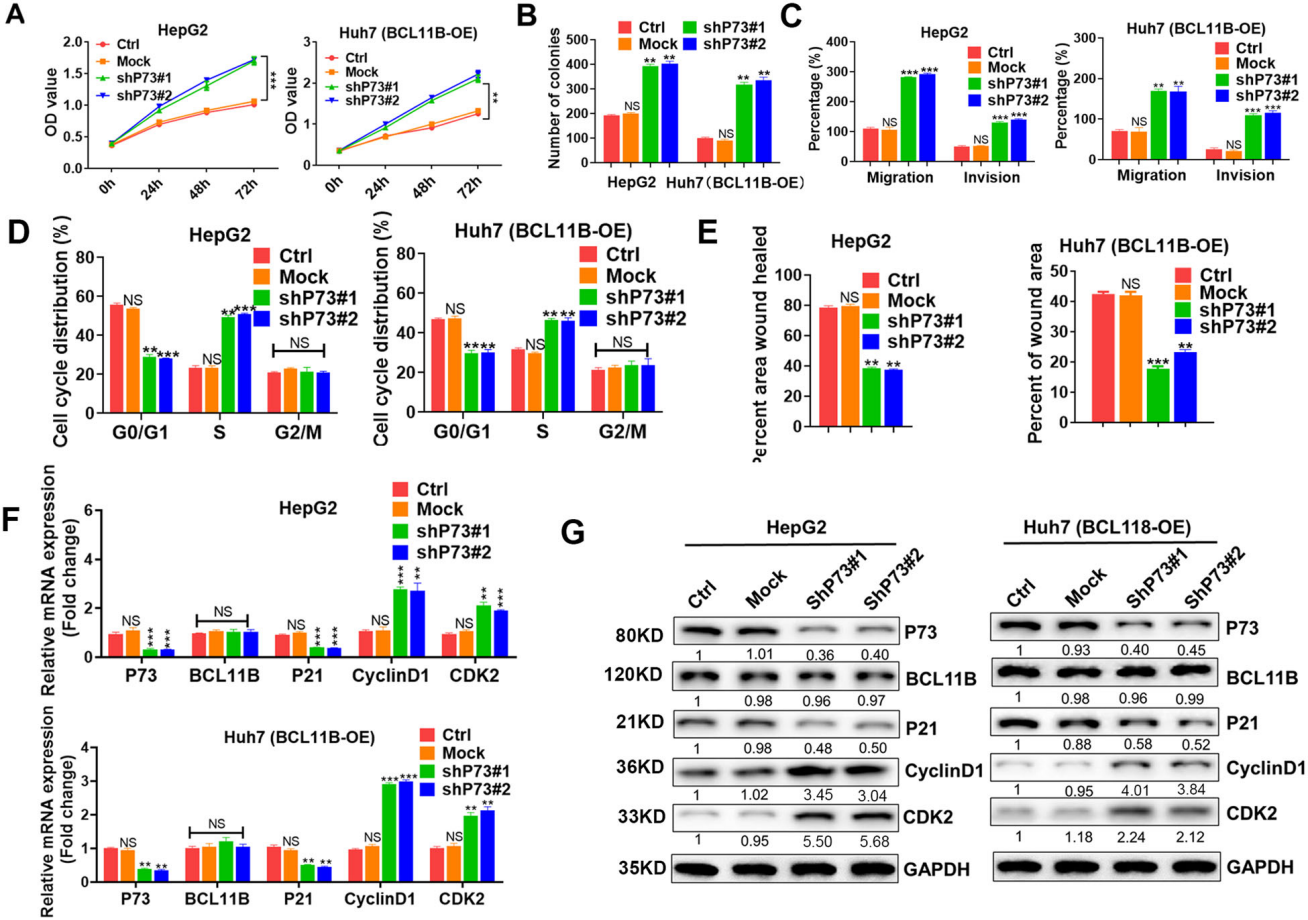
Knockdown P73 greatly abolished inhibitory effects of BCL11B expression on cell proliferation and cell mobility. **a** Effects of P73 silencing on cell proliferation capacities were assessed by CCK-8 assays in HepG2 cells and BCL11B-OE Huh7 cells. **b** Effects of P73 silencing on cell proliferation capacities were assessed by clone formation assays in HepG2 cells and BCL11B-OE Huh7 cells. **c** Effects of P73 silencing on cell mobility potentials were assessed by Transwell assays in HepG2 cells and BCL11B-OE Huh7 cells. **d** Effects of P73 silencing on cell cycle were assessed by flow cytometry in HepG2 cells and BCL11B-OE Huh7 cells. **e** Effects of P73 silencing on cell migration potentials were assessed by wound healing assays in HepG2 cells and BCL11B-OE Huh7 cells. **f, g** mRNA expression (**f**) and protein expression (**g**) of P53 downstream targets and cell cycle-related markers were analyzed in P73-silenced HCC cell lines. Corresponding parental cells were set as controls. Means ± SD from three independent experiments are presented; *P < 0.05; **P < 0.01; ***P < 0.001.

We next aimed to confirm the essential role of P73 in the BCL11B-mediated inhibition of HCC cells further. Results showed that P73 knockdown significantly enhanced the proliferation of BCL11B-expressing HepG2 cells and abolished the inhibitory effects on proliferation caused by BCL11B overexpression in Huh7 cells (Fig. 6a, b and Supplementary Fig. 6a). Moreover, P73 knockdown resulted in substantial accumulation of HCC cells in S phase, accompanied by a substantial decrease in the numbers of cells in G0/G1 phase (all *P* < 0.05; Fig. 6d). Knockdown of P73 increased the numbers of migrating and invasive BCL11B-expressing HepG2 cells and rescued the migration and invasion capacity inhibition in BCL11B-overexpressing Huh7 cells (all *P* < 0.01; Fig. 6c, e and Supplementary Fig. 6c, d). P73 knockdown did not affect BCL11B expression but caused the downregulation of P21 but induced CDK2 and Cyclin D1 upregulation in BCL11B-high cells. Notably, P73 knockdown abolished the inhibitory effects of BCL11B overexpression in Huh7 cells (Fig. 6f, g).

### BCL11B exerted inhibitory effects on stemness traits in HCC via P73

**Fig. 7:**
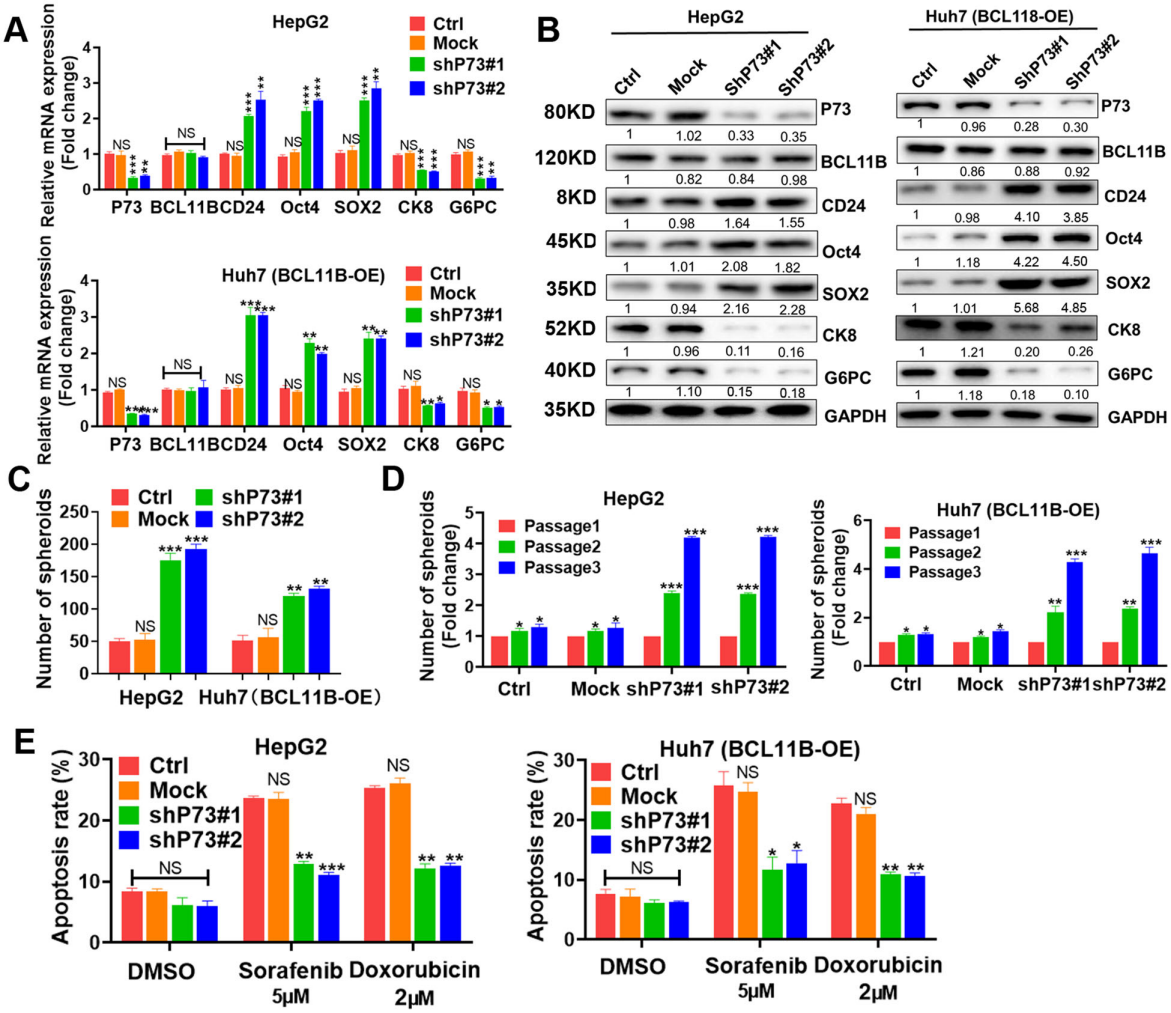
P73 downregulation greatly abolished inhibitory effects of BCL11B expression on cell stemness. **a** mRNA expression levels of CSC-related markers and cell differentiation-related markers were analyzed in P73-silenced HCC cell lines. **b** Protein expression levels of CSC-related markers and cell differentiation-related markers were analyzed in P73-silenced HCC cell lines. **c** Effects of P73 silencing on CSC traits were assessed by sphere-forming assays in HepG2 cells and BCL11B-OE Huh7 cells. **d** Self-renewal ability of P73-silenced HepG2 cells and BCL11B-OE Huh7 cells was evaluated by in vitro serial sphere-forming assays. **e** Cell apoptosis rates of P73-silenced HepG2 cells and BCL11B-OE Huh7 cells were evaluated by flow cytometry after 5 μM sorafenib or 2 μM doxorubicin treatment. Error bars represent the standard deviation (SD) from at least three independent experiments; *,*P* < 0.05; **P < 0.01; ***P < 0.001.

After P73 knockdown, expressions of stemness-associated genes (CD24, OCT4, and SOX2) were increased, whereas those of differentiation-related genes (CK8 and G6PC) were decreased in BCL11B-expressing HepG2 cells. P73 silencing dramatically abolished the regulatory effects caused by ectopic expression of BCL11B (Fig. 7a, b). Accordingly, P73 exert impressive inhibition on CSC-like phenotype, evidenced by increasing numbers of spheroids (Fig. 7c and Supplementary Fig. 6b) and enhanced self-renewal capacities (*P* < 0.01; Fig. 7d) after P73 knockdown. Flow cytometry assays showed that P73 knockdown enhanced drug resistance in HepG2 cells and abolished drug sensitization caused by BCL11B overexpression in Huh7 cells (*P* < 0.01; Fig. 7e). To better clarify the inhibitory functions of BCL11B was related to p73 instead of p53, we also silenced P53 expression in HepG2 cells. Although P53 knockdown resulted in a slighter inhibition of cell proliferation, mobility, and stemness, these effects were not compatible to those of BCL11B knockdown. However, P73 knockdown resulted in almost the same inhibition effects as BCL11B silence did (Supplementary Fig. 7a–h). Collectively, our data demonstrated that P73, but not P53, might be the key downstream molecule of BCL11B-mediated inhibitory effects observed in HCC cells.

## Discussion

A better understanding of CSCs will provide novel insights into HCC carcinogenesis, with potentially major therapeutic implications. Our data identified BCL11B as a novel suppressor of liver CSCs. We found that BCL11B was universally downregulated in HCC tissues. Ectopic expression of BCL11B in HCC cells resulted in inhibitory effects on proliferation, cell cycle progression, and cell mobility. BCL11B significantly hindered liver CSC traits and promoted HCC cell differentiation. Further investigations revealed that BCL11B could trigger the transcription of P73, and resulted in subsequent suppression function in HCC. Overall, our data indicated BCL11B as a promising therapeutic target for HCC with the potential to eradicate CSCs.

BCL11B was previously considered as a haploinsufficient suppressor of tumorigenesis^28,29^, but its role in regulating stemness traits remained controversial. A recent study revealed BCL11B as a key regulator of T-cell differentiation and maturation^30^. Attenuation of BCL11B activity also greatly enhanced β-catenin signaling, indicating a repressive effect of BCL11B in the carcinogenesis of colorectal cancers^22,31^^.^ However, it was also reported that enriched BCL11B expression in highly tumorigenic glioma cells promoted cancer cell growth by regulating the expression of stemness-associated genes (SOX2 and BMI1)^32^. Furthermore, high BCL11B expression was necessary for mammary epithelial cells to maintain quiescence^33^. The distinct and contrasting functions of BCL11B in different cancers might be attributed to the complexity and heterogeneity of CSCs. It indicates that various CSC models might be responsible for BCL11B function in different cancers^34–36^. Additionally, it was reported that CSCs were derived from normal stem cells, and the accumulation of genetic or epigenetic mutations along with the effects of the tumor microenvironment caused the CSCs to become highly heterogeneous^37^. We showed that BCL11B could greatly disrupt spheroid formation and stemness-associated gene expression in HCC. In vitro experiments identified BCL11B as a critical molecule for the sensitization of HCC cells to sorafenib and doxorubicin. Our findings are in line with previous studies of colorectal cancers and clearly identify BCL11B as an inhibitor of CSC traits in HCC^22,31^.

P53 signaling is a vital pathway that is suppressed in various types of cancer because of its regulatory roles in diverse biological functions such as cell cycle progression, proliferation, migration, invasion, apoptosis, and senescence^38^. Negative correlations between P53 signaling and CSC characteristics are frequently observed in solid tumors, including breast cancer^39^, lung carcinoma^40^, prostate cancer^41^, and colorectal cancer^42^. In HCC, it is reported that P53 signaling interferes with CSC self-renewal and stemness by inhibiting the expression of stemness-associated genes such as NANOG^18^. Our previous investigation revealed that P53 mutation occurs in almost 60% of Chinese patients with HBV-related HCC, suggesting that mutation of the P53 gene not only abolishes the P53 tumor-suppressor function but also endows the mutant P53 protein with a gain-of-function to promote tumorigenesis^16^. Considering the biological significance of P53 signaling, it is an appealing strategy to reactivate P53 signaling in patients harboring mutant P53 proteins, which might provide survival benefits for patients with HBV-related HCC.

The transcription factor P73 was previously reported to have similar functions as wild-type P53. It can bind to the regulatory regions of P53 target genes, thereby inducing cell death and enhancing cell growth. P73 has an extremely low mutation frequency in solid tumors^24,43,44^ and is therefore considered a potential target for restoring P53 signaling, especially in patients with P53 mutation. Our data revealed that BCL11B is essential for triggering P73 transcription and that P73 serves as a key downstream target through which BCL11B exerts its inhibitory functions in HCC. Critically, the silencing of P73 abolished the inhibitory effects of BCL11B overexpression. Our results suggest that the upregulation of P73 expression might be a way to reactivate P53 signaling in HCC. Considering that various other strategies have failed to restore P53 signaling in HCC, our findings provide an alternative way to enhance P53 signaling by inducing the upstream regulator BCL11B, which may have the potential to hinder disease progression and CSC traits in HCC. It remains unclear, however, how BCL11B triggers P73 transcription. Further investigation will be required to fully explore that question.

In summary, our results emphasize the clinical value as well as the biological function of BCL11B in HCC. We identified BCL11B as a novel tumor suppressor in HCC that exerts inhibitory effects on cell proliferation and migration. BCL11B enhanced the chemosensitivity of HCC cells, attenuating their self-renewal, and promoting their differentiation. BCL11B exerted its function mainly by upregulating P73 expression to reactivate P53 signaling, suggesting an exciting target for the reactivation of wild-type P53 signaling in individuals harboring P53 mutations. Restoration of BCL11B expression might be an attractive strategy for HCC therapy.

## Materials and methods

### Patients samples

Two independent cohorts of patients with HCC were recruited from Zhongshan Hospital, Fudan University. The first cohort consisted of 20 pairs of HCC samples and matched adjacent non-tumor tissue samples collected in 2018. The second cohort included 189 patients with HCC who underwent surgical resection between 2012 and 2013. The specific sample information was shown in supplementary materials and methods. The Human Research Ethics Committee of Zhongshan Hospital, Fudan University approved the study protocol. All patients provided written, informed consent for inclusion in the study.

### In vivo assay

Male nude mice (4 weeks old) were purchased from the Department of Experimental Animals of the Chinese Academy of Sciences (Shanghai, China). Animal care and experimental protocols were conducted under guidelines approved by the Institutional Animal Care and Use Committee (IACUC) at Zhongshan Hospital, Fudan University. MHCC97L cells (untransfected, transfected with empty [Mock] lentivirus vector, or transfected with BCL11B-specific short-hairpin [sh] RNA) and Huh 7 cells (untransfected, transfected with empty vector [NC], or transfected with a BCL11B-overexpression construct) were suspended in 200 μl serum-free DMEM and Matrigel (1:1). The cells were then injected orthotopically into the livers of nude mice for proliferation analysis (5 × 10^6^ cells per mouse). For analysis of the self-renewal capacity of HCC cells in vivo, nude mice were injected subcutaneously with serially diluted (total of 10^2^, 10^3^, 10^4^, or 10^5^ cells) BCL11B-knockdown MHCC97L cells or BCL11B-overexpressing Huh 7 cells. After 6 weeks, the resulting tumors were detached from the mice, weighed, and photographed.

### Statistics

SPSS 18.0 and Graph Pad were used for statistical analysis. All data were shown as the mean ± standard error of the mean (SEM). *P* < 0.05 was considered statistically significant. Data were tested for statistical significance by paired two-tailed t-test. Categorical data were compared by *Χ*^2^ test and Fisher’s exact test. OS and TTR curves were analyzed by the Kaplan–Meier method and the log-rank test. Cox proportional hazards regression models were constructed to perform univariate and multivariate analyses of prognostic parameters. *P* values of statistical significance are shown in the respective figures.

## Supplementary information

Supplementary Figure and Table legends

Supplementary Figure 1

Supplementary Figure 2

Supplementary Figure 3

Supplementary Figure 4

Supplementary Figure 5

Supplementary Figure 6

Supplementary Figure 7

Supplementary Table 1

Supplementary Table 2

Supplementary Table 3

Supplementary Table 4

Supplementary Table 5

Supplementary materials and methods
