# Supplementary material for: BCL11B suppresses tumor progression and stem cell traits in hepatocellular carcinoma by restoring p53 signaling activity
Source: Cell Death Dis. 2020 Oct 22;11(10):895. doi: 10.1038/s41419-020-03115-3 (PMC7581528; doi:10.1038/s41419-020-03115-3)
Supplement: Supplementary file 1 — Supplementary Figure and Table legends [file 41419_2020_3115_MOESM1_ESM.docx]

**BCL11B suppresses tumor progression and stem cell traits in hepatocellular carcinoma by restoring p53 signaling activity**

Wen-Jing Yang1*, Yun-Fan Sun 2*, An-Li Jin1, Li-Hua Lv1, Jie Zhu1, Bei-Li Wang1, Zhou Yan1, Chun-Yan Zhang1, Hao Wang 1, Bo Hu2, Peng-Xiang Wang2, Te Liu3, Bai-Shen Pan1, Jian Zhou2, Jia Fan2, Xin-Rong Yang2*, Wei Guo1*

1. Department of Laboratory Medicine, Zhongshan Hospital, Fudan University, Shanghai, China;

2. Department of Liver Surgery & Transplantation, Liver Cancer Institute, Zhongshan Hospital, Fudan University; Key Laboratory of Carcinogenesis and Cancer Invasion, Ministry of Education, Shanghai 200032, P. R. China.

3. Shanghai Geriatric Institute of Chinese Medicine, Shanghai University of Traditional Chinese Medicine, Shanghai, 200031, China.

**Supplementary Figure and Table legends**

Supplementary Figure 1. BCL11B inhibits proliferation, induces G0/G1 arrest and attenuates mobility.

**(A)** The TCGA database shows that BCL11B expression was dramatically decreased in LIHC cohort. **(B)** HCC patients with low BCL11B expression had a significantly poorer prognosis. **(C)** BCL11B expression in 8 cell lines was evaluated by qRT-PCR and WB assays. **(D)** MHCC97L cells were successfully transfected with shRNAs against BCL11B, and BCL11B was successfully overexpressed in Huh7 cells. **(E)** Graphs of cell proliferation detected by colony formation assay in the indicated cells. **(F)** Graphs of cell cycle detected by flow cytometry in the indicated cells. **(G)** Graphs of cell migration and cell invasion detected by transwell assay in the indicated cells. **(H)** Graphs of cell migration detected by wound healing assay in the indicated cells.

Supplementary Figure 2. BCL11B inhibits cell self-renewal.

**(A)** mRNA expression of CSC related markers in shBCL11B cells (MHCC97L and HepG2). **(B)** mRNA expression of CSC related markers in BCL11B-OE cells (Huh7). **(C)** Graphs of spheroid formation ability in the indicated assay.

Supplementary Figure 3. Knockdown BCL11B in L02 cells enhanced cell cell mobility and cell stemness.

**(A)** RT-PCR showed the mRNA expression of the indicated markers. **(B)** WB assays showed the protein expression of the indicated markers. **(C)** Spheroid formation assays showed the number of spheroid cells. **(D)** Transwell assays showed the cell migration and invision of the indicated plasmids. **(E)** CCK8 assays showed the cell proliferation of the indicated plasmids.

Supplementary Figure 4. BCL11B enhances cell chemosensitivity.

**(A)** Graphs of cell apoptosis rate respond to sorafenib and doxorubicin treatment detected by flow cytometry in the indicated cells. **(B)** Graphs of cell survival rate respond to sorafenib and doxorubicin treatment detected by flow cytometry in the indicated cells.

Supplementary Figure 5. BCL11B enhances cell differentiation.

**(A)** Expression levels of CSC markers (EpCAM and CD24) and cell differentiation markers (CK8 and GP6C) in the indicated cells were analyzed in a time-dependent manner via WB assay. **(B)** Graphs of CD24^+^ and CK8^+^ cell percentage in the indicated cells.

Supplementary Figure 6. Knockdown P73 rescued BCL11B-induced inhibitory effects on cell proliferation, cell mobility and cell self-renewal.

**(A)** Graphs of cell proliferation detected by colony formation assay in the indicated cells. **(B)** Graphs of spheroid formation ability in the indicated assay. **(C)** Graphs of cell migration and cell invasion detected by transwell assay in the indicated cells. **(D)** Graphs of cell migration detected by wound healing assay in the indicated cells.

Supplementary Figure 7. BCL11B enhances cell CSC traits in a P73 dependent manner, but not P53-dependent.

**(A)** RT-PCR showed the mRNA expression of the indicated markers. **(B)** WB assays showed the protein expression of the indicated markers. **(C)** Huh7 cells were successfully transfected with siRNAs against E2F1. **(D)** CCK8 assays showed the cell proliferation of the indicated plasmids. **(E)** Transwell assays showed the cell migration and invision of the indicated plasmids. **(F)** Spheroid formation assays showed the number of spheroid cells. **(G)** Flow cytometry assays showed the drug chemoresistance of the indicated cells. **(H)** Colony formation assays showed the drug chemoresistance of the indicated cells.

Supplementary Table 1.

The correlations between BCL11B expression and clinicopathological characteristics in HCC.

Supplementary Table 2.

Univariate cox proportional regression analysis of factors associated with recurrence and overall survival

Supplementary Table 3.

The targeting sequences of small hairpin RNAs were used in study.

Supplementary Table 4.

The primer sequences were used in study.

Supplementary table S5.

All antibodies were used in study.
