# Supplementary figures and images for: BCL11B suppresses tumor progression and stem cell traits in hepatocellular carcinoma by restoring p53 signaling activity
Source: Cell Death Dis. 2020 Oct 22;11(10):895. doi: 10.1038/s41419-020-03115-3 (PMC7581528; doi:10.1038/s41419-020-03115-3)

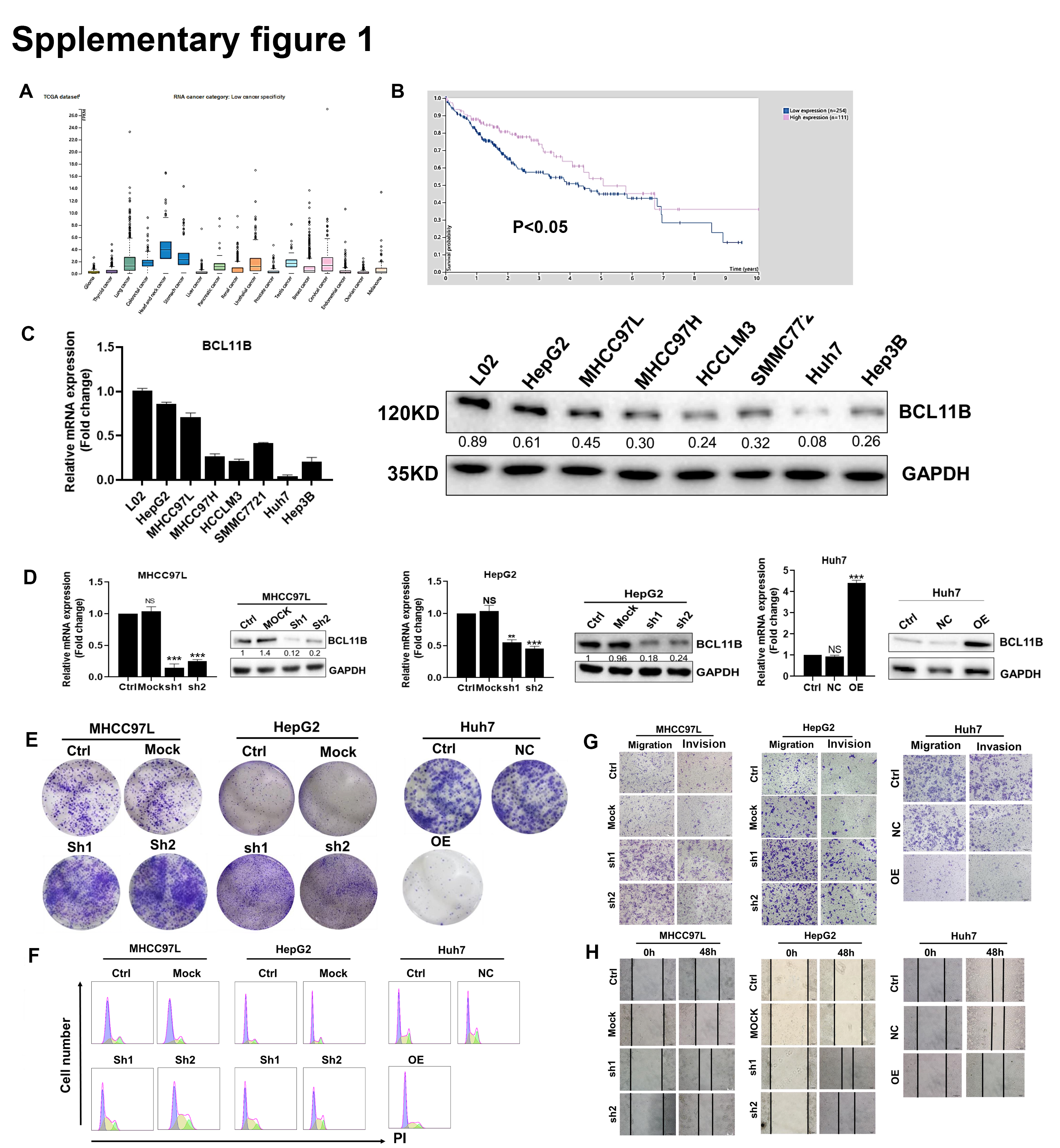

Supplement: Supplementary file 2 — Supplementary Figure 1 [file 41419_2020_3115_MOESM2_ESM.tif]

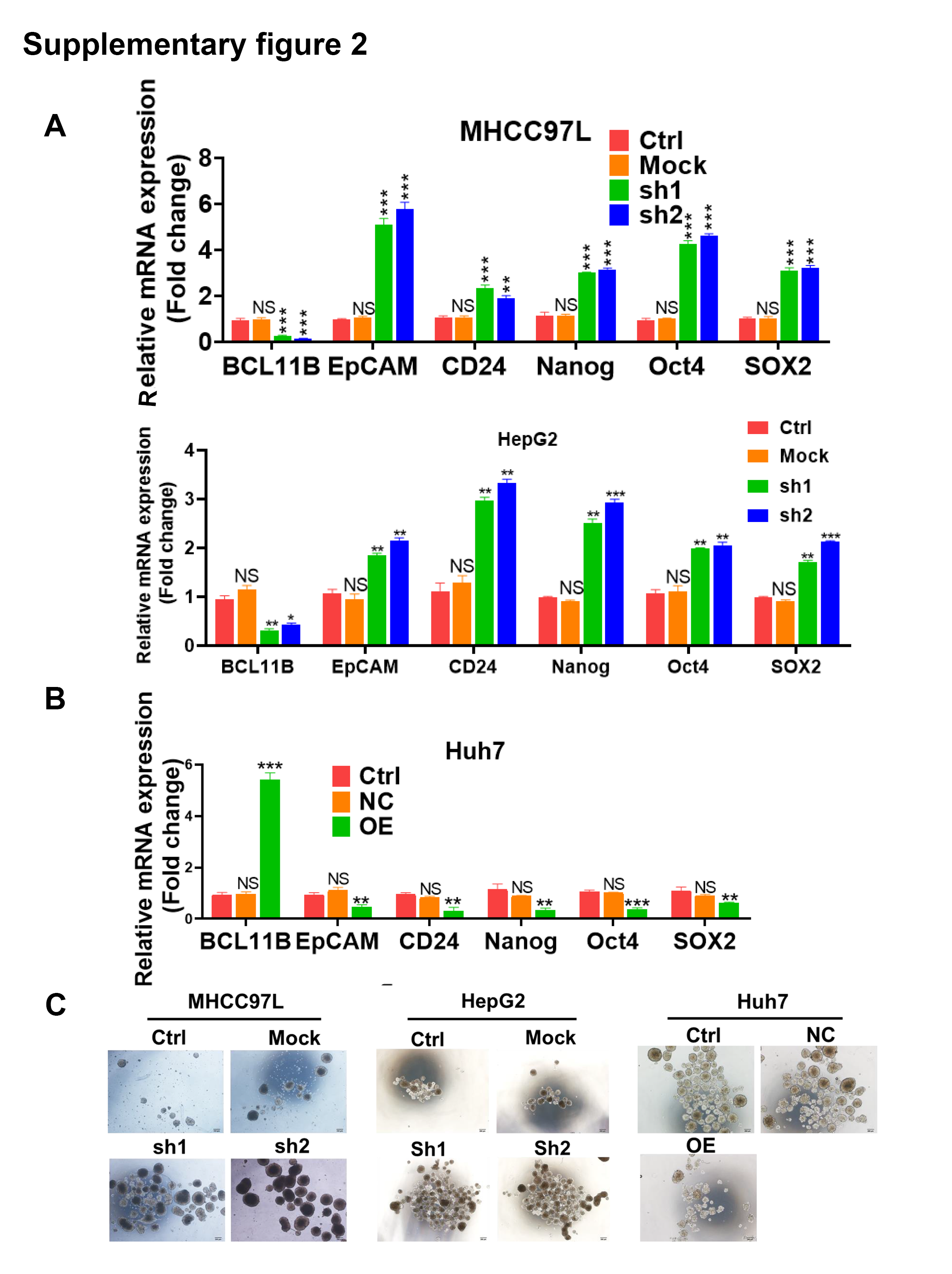

Supplement: Supplementary file 3 — Supplementary Figure 2 [file 41419_2020_3115_MOESM3_ESM.tif]

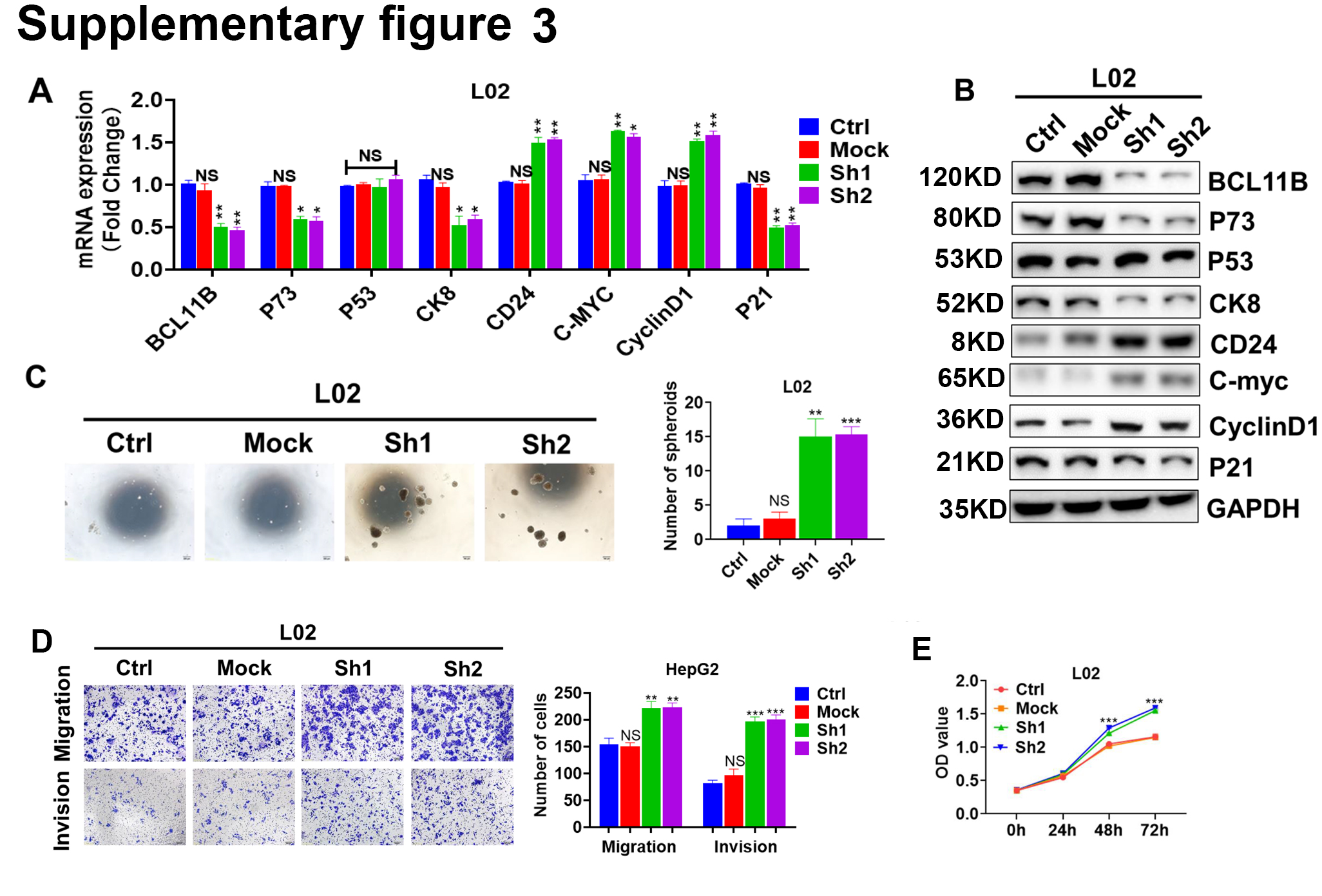

Supplement: Supplementary file 4 — Supplementary Figure 3 [file 41419_2020_3115_MOESM4_ESM.tif]

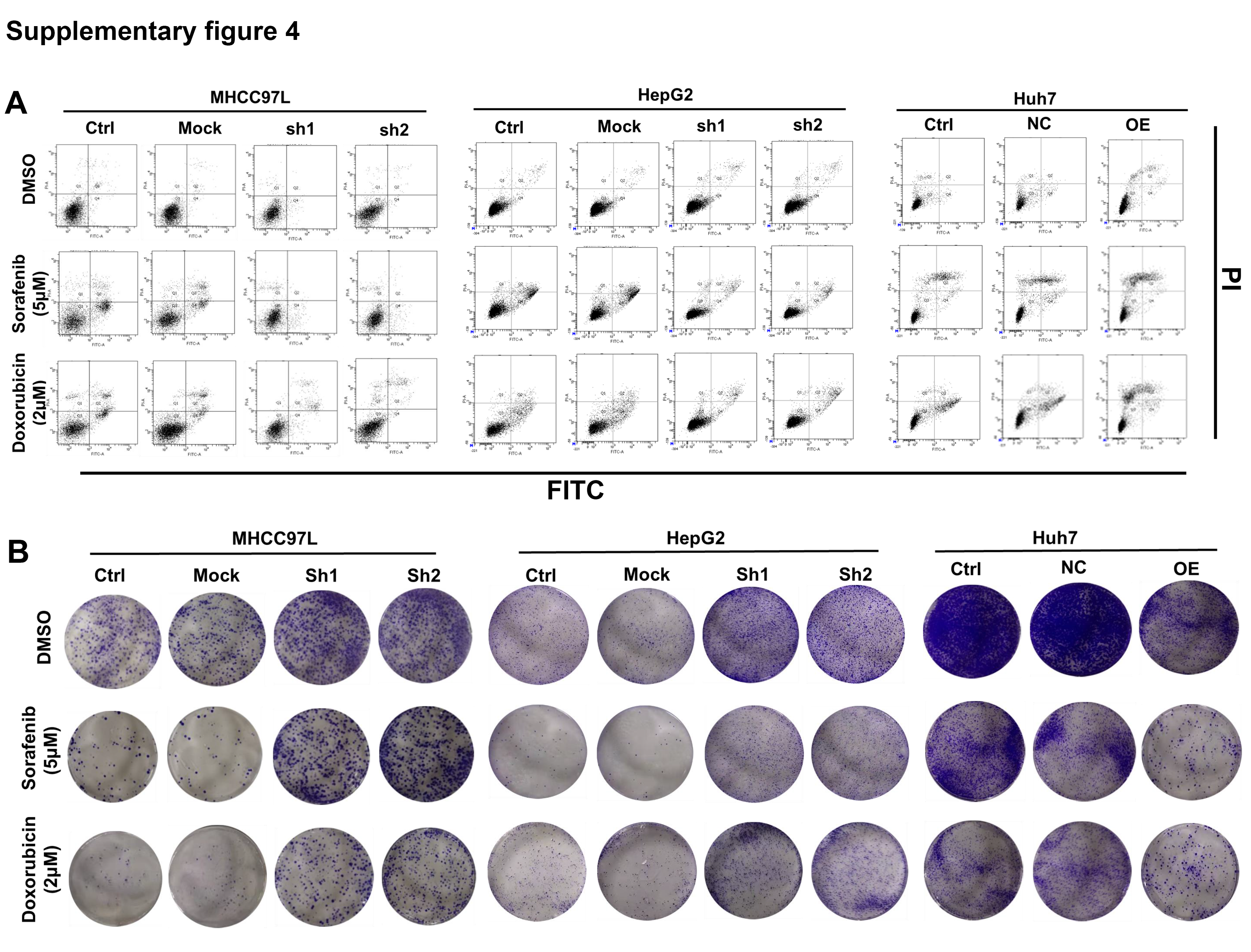

Supplement: Supplementary file 5 — Supplementary Figure 4 [file 41419_2020_3115_MOESM5_ESM.tif]

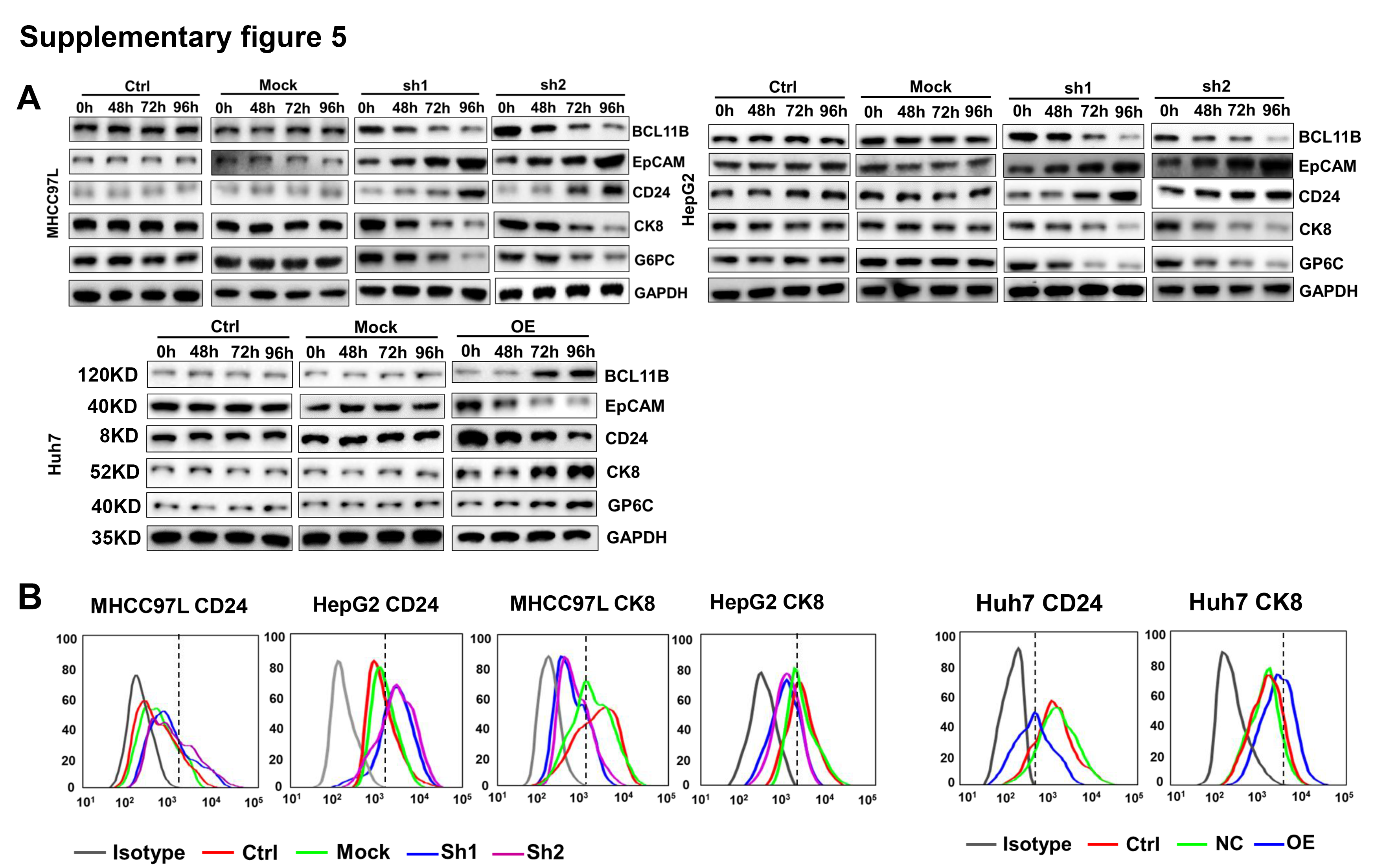

Supplement: Supplementary file 6 — Supplementary Figure 5 [file 41419_2020_3115_MOESM6_ESM.tif]

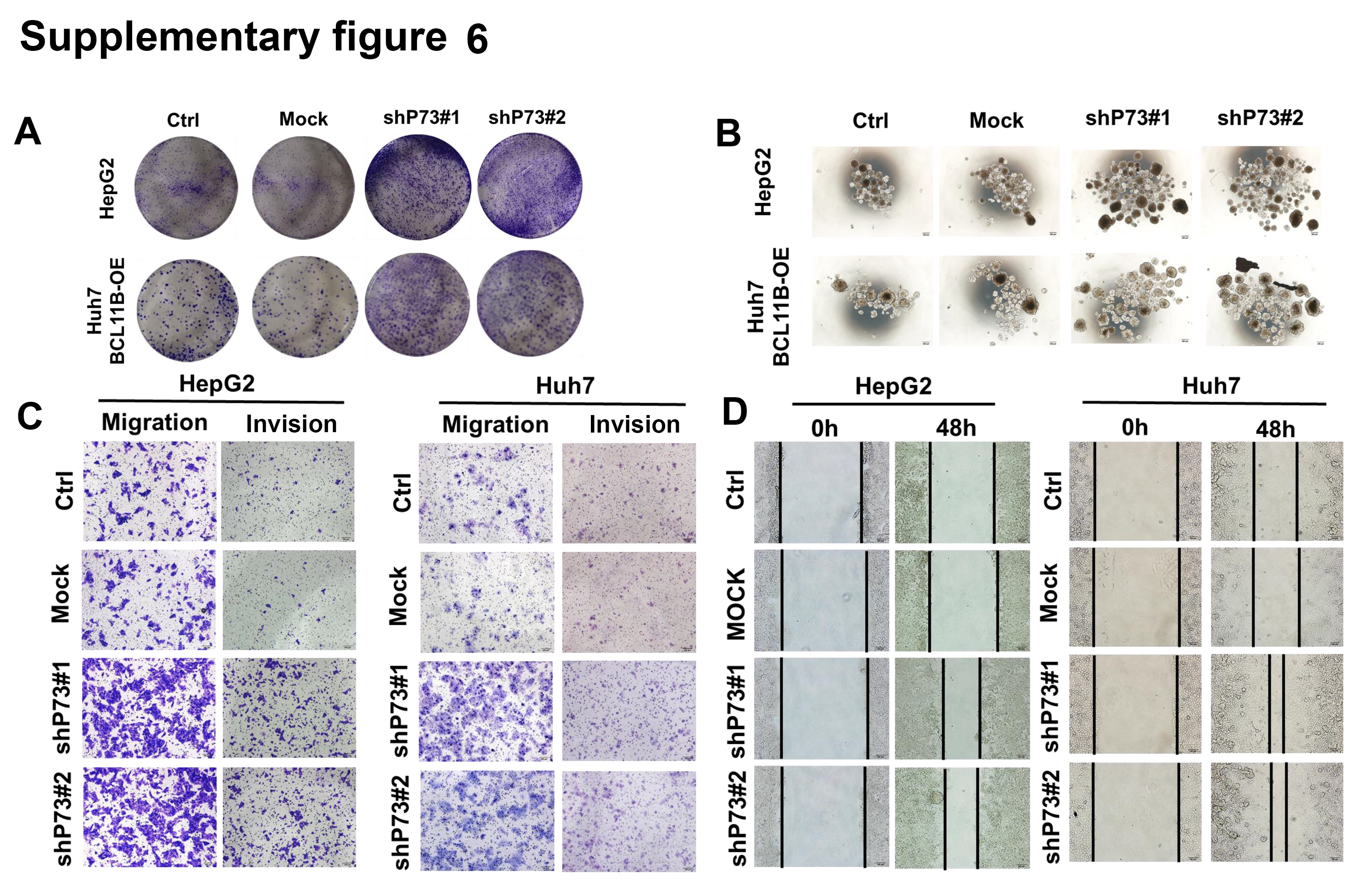

Supplement: Supplementary file 7 — Supplementary Figure 6 [file 41419_2020_3115_MOESM7_ESM.tif]

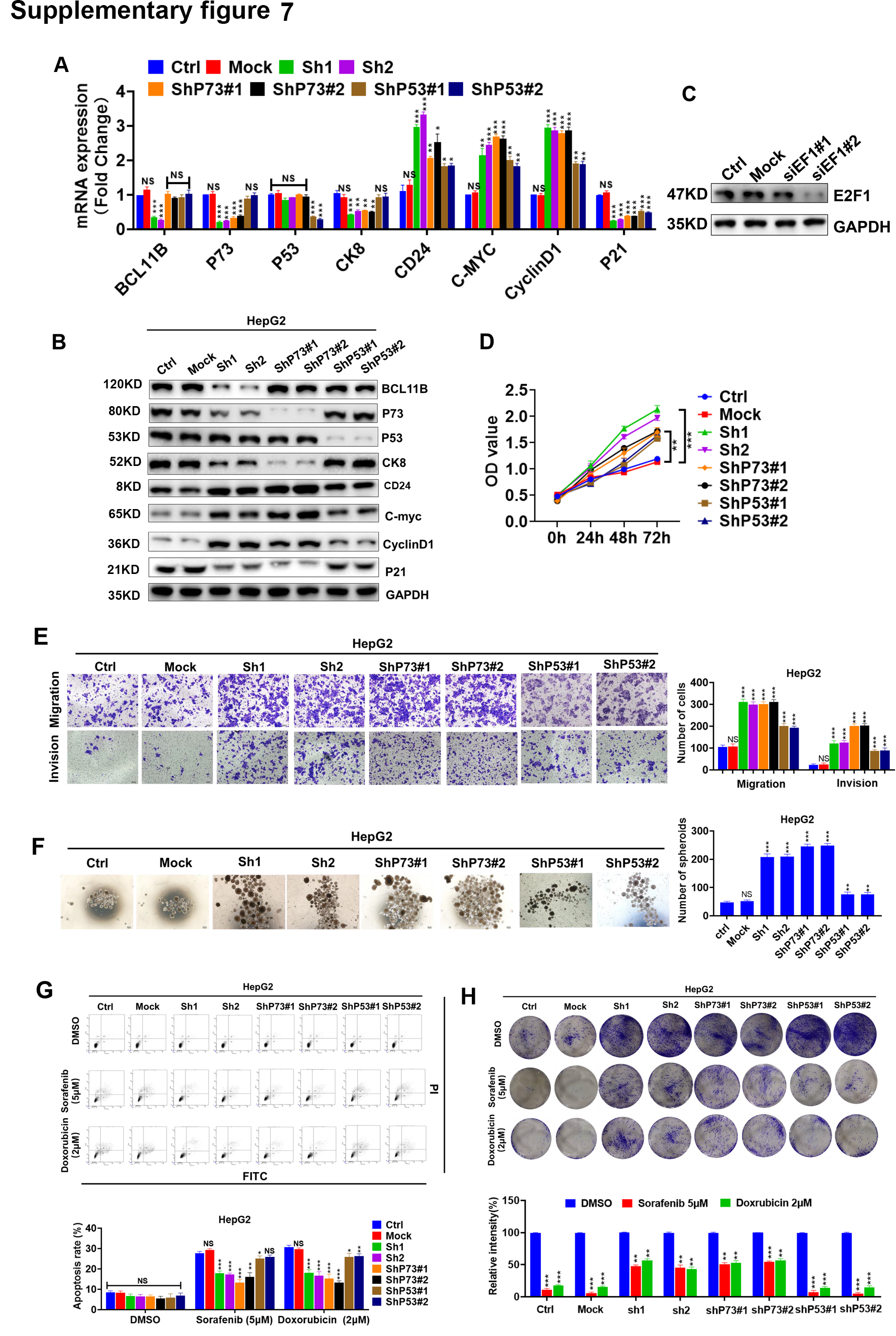

Supplement: Supplementary file 8 — Supplementary Figure 7 [file 41419_2020_3115_MOESM8_ESM.tif]
