# Supplementary material for: BCL11B suppresses tumor progression and stem cell traits in hepatocellular carcinoma by restoring p53 signaling activity
Source: Cell Death Dis. 2020 Oct 22;11(10):895. doi: 10.1038/s41419-020-03115-3 (PMC7581528; doi:10.1038/s41419-020-03115-3)
Supplement: Supplementary file 9 — Supplementary Table 1 [file 41419_2020_3115_MOESM9_ESM.docx]

| **Supplementary Table 1.** The correlations between BCL11B expression and clinicopathological characteristics in HCC. | | | | | |
| --- | --- | --- | --- | --- | --- |
|  | |  | **BCL11B expression** | | **P** |
|  |  |  | **Low (127)** | **high (62)** |  |
| Gender | Male | 151 | 101 (66.9) | 50 (33.1) | 0.857 |
|  | Female | 38 | 26 (68.4) | 12 (31.6) |  |
| Age (years) | >50 | 110 | 77 (70.0) | 33 (30.0) | 0.333 |
|  | ≤50 | 79 | 50 (63.3) | 29 (36.7) |  |
| Child-Pugh score | A | 179 | 121 (67.6) | 58 (32.4) | 0.618 |
|  | B | 10 | 6 (60.0) | 4 (40.0) |  |
| Liver cirrhosis | No | 42 | 28 (66.7) | 14 (33.3) | 0.934 |
|  | Yes | 147 | 99 (67.3) | 48 (32.7) |  |
| ALT,U/L | ≤40 | 134 | 90 (67.2) | 44 (32.8) | 0.988 |
|  | >40 | 55 | 37 (67.3) | 18 (32.7) |  |
| AST,U/L | ≤40 | 135 | 87 (64.4) | 48 (35.6) | 0.203 |
|  | >40 | 54 | 40 (74.1) | 14 (25.9) |  |
| AFP, ng/ml | ≤400 | 140 | 98 (70.0) | 42 (30.0) | 0.165 |
|  | >400 | 49 | 29 (59.2) | 20 (40.8) |  |
| Tumor number | Single | 169 | 112 (66.3) | 57 (33.7) | 0.432 |
|  | Multiple | 20 | 15 (75.0) | 5 (25.0) |  |
| Tumor size, cm | ≤5 | 119 | 77 (64.7) | 42 (35.3) | 0.342 |
|  | >5 | 70 | 50 (71.4) | 20 (28.6) |  |
| Tumor encapsulation | Complete | 123 | 82 (66.7) | 41 (33.3) | 0.832 |
|  | None | 66 | 45 (68.2) | 21 (31.8) |  |
| Satellite lesion | No | 171 | 115 (67.3) | 56 (32.7) | 0.960 |
|  | Yes | 18 | 12 (66.7) | 6 (33.3) |  |
| Vascular invasion | No | 107 | 67 (62.6) | 40 (37.4) | 0.126 |
|  | Yes | 82 | 60 (73.2) | 22 (26.8) |  |
| Edmondson stage | I-II | 126 | 77 (61.1) | 49 (38.9) | **0.012** |
|  | III-IV | 63 | 50 (79.4) | 13 (20.6) |  |
| BCLC stage | 0+A | 158 | 101 (63.9) | 57 (36.1) | **0.031** |
|  | B+C | 31 | 26 (83.9) | 5 (16.1) |  |
| Chinese liver tumor stage | I-II | 179 | 121(67.6) | 58(32.4) | 0.618 |
|  | III-IV | 10 | 6(60.0) | 4(40.0) |  |
| Abbreviations: ALT, alanine aminotransferase; AST, aspartate transaminase; AFP, α-fetoprotein; BCLC, Barcelona Clinic Liver Cancer. | | | | | |
