# Supplementary material for: BCL11B suppresses tumor progression and stem cell traits in hepatocellular carcinoma by restoring p53 signaling activity
Source: Cell Death Dis. 2020 Oct 22;11(10):895. doi: 10.1038/s41419-020-03115-3 (PMC7581528; doi:10.1038/s41419-020-03115-3)
Supplement: Supplementary file 10 — Supplementary Table 2 [file 41419_2020_3115_MOESM10_ESM.docx]

| **Supplementary Table 2.** Univariate cox proportional regression analysis of factors associated with recurrence and overall survival | | | | | |
| --- | --- | --- | --- | --- | --- |
| **Variables** | **Recurrence** | |  | **Overall survival** | |
|  | **HR (95% CI)** | **P** |  | **HR(95% CI)** | **P** |
| Age  (>50y versus ≤50y) | 0.73  (0.50-1.07) | 0.104 |  | 0.64  (0.41-1.02) | **0.060** |
| Sex  (male versus female) | 0.76  (0.48-1.21) | 0.249 |  | 1.16  (0.69-1.95) | 0.563 |
| Liver cirrhosis  (yes versus no) | 1.52  (0.92-2.49) | 0.099 |  | 1.11  (0.63-1.96) | 0.712 |
| ALT  (>40U/L versus ≤40U/L) | 1.57  (1.06-2.34) | **0.025** |  | 1.80  (0.85-2.24) | 0.190 |
| AST  (>40U/L versus ≤40U/L) | 1.78  (1.20-2.64) | **0.004** |  | 1.49  (0.92-2.41) | 0.101 |
| AFP  (>400ng/ml versus ≤400ng/ml) | 1.86  (1.25-2.78) | **0.002** |  | 1.49  (0.91-2.43) | 0.117 |
| No. of tumors  (multi versus single) | 1.81  (1.06-3.08) | **0.030** |  | 1.57  (0.84-3.02) | 0.157 |
| Tumor size  (>5cm versus ≤5cm) | 2.35  (1.60-3.43) | **<0.001** |  | 1.59  (0.99-2.48) | 0.056 |
| Tumor encapsulation  (none versus complete) | 1.15  (0.78-1.69) | 0.452 |  | 1.03  (0.64-1.67) | 0.903 |
| Satellite lesions  (yes versus no) | 1.67  (0.96-2.88) | 0.069 |  | 0.78  (0.33-1.76) | 0.528 |
| vascular invasion  (yes versus no) | 2.08  (1.42-3.04) | **<0.001** |  | 1.91  (1.21-3.04) | **0.006** |
| Edmondson stage  (III-IV versus I-II) | 1.78  (1.21-2.61) | **0.003** |  | 1.50  (0.94-2.39) | 0.087 |
| BCLC stage  (B+C versus 0+A) | 1.98  (1.27-3.09) | **0.003** |  | 1.72  (1.01-2.94) | **0.045** |
| Chinese liver tumor stage  (III-IV versus I-II) | 2.38  (1.24-4.57) | **0.009** |  | 1.72  (0.75-3.95) | 0.203 |
| BCL11B  (Low versus high) | 2.55  (1.42-4.57) | **0.015** |  | 2.21  (1.27-3.85) | **0.002** |
| Abbreviations: ALT, alanine aminotransferase; AST, aspartate transaminase; AFP, α-fetoprotein; BCLC, Barcelona Clinic Liver Cancer; HR, hazard ratio. | | | | | |
