# Supplementary material for: BCL11B suppresses tumor progression and stem cell traits in hepatocellular carcinoma by restoring p53 signaling activity
Source: Cell Death Dis. 2020 Oct 22;11(10):895. doi: 10.1038/s41419-020-03115-3 (PMC7581528; doi:10.1038/s41419-020-03115-3)
Supplement: Supplementary file 11 — Supplementary Table 3 [file 41419_2020_3115_MOESM11_ESM.docx]

**Supplementary Table 3.** The targeting sequences of small hairpin RNAs were used in study.

| Gene | sequences |
| --- | --- |
| shRNA#1 against p73 | Sense; 5′-ATCCGCGTGGAAGGCAATAAT-3′  Anti-sense: 5′-ATTATTGCCTTCCACGCGGAT-3′ |
| shRNA#2 against p73 | Sense: 5′-CTGTCATGGCCCAGTTCAATC-3′  Anti-sense: 5′-GATTGAACTGGGCCATGACAG-3′ |
| shRNA#1 against BCL11B | 5‘AAAAGTCCCAAGCAGGAGAACATTTGGATCCAAATGTTCTCCTGCTTGGGAC-3’ |
| shRNA#2 against BCL11B | 5‘AAAAGAGCCTTCCAGCTACATTTGTTGGATCCAACAAATGTAGCTGGAAGGCTC-3’ |
| Scrambled shRNA | 5’GCAGTTATCTGGAAGATCAGGTTGGATCCAACCTGATCTTCCAGATAACTGC-3’ |
| SiE2F1  Ctrl | 5’-GACCACCUGAUGAAUAUCUTT-3’  5’-AGAUAUUCAUCAGGUGGUCTT-3’  5’-UUCUCCGAACGUGUCACGUTT-3’  5’-ACGUGACACGUUCGGAGAATT-3’ |
