# Supplementary material for: BCL11B suppresses tumor progression and stem cell traits in hepatocellular carcinoma by restoring p53 signaling activity
Source: Cell Death Dis. 2020 Oct 22;11(10):895. doi: 10.1038/s41419-020-03115-3 (PMC7581528; doi:10.1038/s41419-020-03115-3)
Supplement: Supplementary file 12 — Supplementary Table 4 [file 41419_2020_3115_MOESM12_ESM.docx]

**Supplementary Table 4.** The primer sequences were used in study.

| Gene |  |  | Sequences |
| --- | --- | --- | --- |
| BCL11B |  | Forward  Reverse | 5′-AGGAGAGTATCTGAGCCAGTG -3′  5′-GTTGTGCAAATGTAGCTGGAAG -3′ |
| GAPDH |  | Forward  Reverse | 5′-GTCATCCAACGGGAATGCA-3′  5′-TGATCGGTTACCGTGATCAAAA-3′ |
| EpCAM  CD24  Nanog  OCT4  SOX2  P53 |  | Forward  Reverse  Forward  Reverse  Forward  Reverse  Forward  Reverse  Forward  Reverse  Forward  Reverse | 5’-GAATAATAATCGTCAATGCCAGTG-3’  5’-CGCTCTCATCGCAGTCAG-3’  5’- CCCACGCAGATTTATTCCAG-3’  5’- ACCACGAAGAGACTGGCTGT-3’  5’-TTTGTGGGCCTGAAGAAAACT-3’  5’-AGGGCTGTCCTGAATAAGCAG-3’  5’-CTGGGTTGATCCTCGGACCT-3’  5’-CCATCGGAGTTGCTCTCCA-3’  5’-GCCGAGTGGAAACTTTTGTCG-3’  5’-GGCAGCGTGTACTTATCCTTCT-3’  5′-CACGAGCTGCCCCCAGG-3′  5′-TCAGTCGACGTCTGAGT-3′ |
| P73 |  | Forward | 5′-GACGGACGCCGATG-3′ |
| P21  c-myc  CDK2  CyclinD1  CK8  G6PC |  | Reverse  Forward  Reverse  Forward  Reverse  Forward  Reverse  Forward  Reverse  Forward  Reverse  Forward  Reverse | 5′-CTGGTCCATGGTGCTG-3′  5′-CCGGCGAGGCCGGGATGAG-3′  5′- CTTCCTCTTGGAGAAGATC-3′  5’-GGCTCCTGGCAAAAGGTCA-3’  5’-CTGCGTAGTTGTGCTGATGT-3’  5' GCCAGAAACAAGTTGACGG3'  5' ATGAGGGGAAGAGGAATGC3'  5' CCCTCGGTGTCCTACTTCA3'  5' CTCCTCGCACTTCTGTTCCT3'  5’-CCTCCACTCCTGCCTCTAC-3’  5’-TGCTGCTGCCCACTCG-3’  5’-TTCTCATTACCTTCTTCCTGTTC-3’  5’-CACCTCTGGGCTTTCTCC-3’ |
