# Supplementary material for: BCL11B suppresses tumor progression and stem cell traits in hepatocellular carcinoma by restoring p53 signaling activity
Source: Cell Death Dis. 2020 Oct 22;11(10):895. doi: 10.1038/s41419-020-03115-3 (PMC7581528; doi:10.1038/s41419-020-03115-3)
Supplement: Supplementary file 13 — Supplementary Table 5 [file 41419_2020_3115_MOESM13_ESM.docx]

**Supplementary table S5.** All antibodies were used in study.

| **Protein** | **corporation** | **Product code** | **KD** |
| --- | --- | --- | --- |
| BCL11B | Santu Cruz | sc-365320 | 120KD |
| GAPDH | Beyotime | AF1186 | 35KD |
| EpCAM | CST | 36746S | 40KD |
| CD24 | abcam | ab179821 | 8KD |
| CD24 | abcam | ab31622 | NA |
| Nanog | CST | 4903S | 42KD |
| Oct4 | Abcam | ab181557 | 45KD |
| SOX2 | CST | 3579S | 35KD |
| c-Myc | CST | 5605S | 65KD |
| CK8 | Abcam | ab53280 | 52KD |
| G6PC | Abcam | ab83690 | 40KD |
| P73 | CST | 14620S | 80KD |
| P53 | CST | 2527S | 53KD |
| P21 | CST | 2947S | 21KD |
| CDK2 | CST | 2546S | 33KD |
| CyclinD1  E2F1 | CST  Abcam | 2978S  Ab245308 | 36KD  47KD |
