# Supplementary material for: BCL11B suppresses tumor progression and stem cell traits in hepatocellular carcinoma by restoring p53 signaling activity
Source: Cell Death Dis. 2020 Oct 22;11(10):895. doi: 10.1038/s41419-020-03115-3 (PMC7581528; doi:10.1038/s41419-020-03115-3)
Supplement: Supplementary file 14 — Supplementary materials and methods [file 41419_2020_3115_MOESM14_ESM.docx]

**Supplementary materials and methods**

**Tissue samples**

The samples from the fist cohort were evaluated for BCL11B expression individually, whereas the samples from the second cohort were used to construct tissue microarrays (TMAs) for BCL11B prognostic evaluation. HCC was confirmed by histological examination in each patient. Patients with other liver diseases or a history of radiotherapy or chemotherapy before surgical resection were excluded. Complete clinical data and follow-up information were gathered for each patient. OS was defined as the length of time between surgery and death. TTR was defined as the length of time between surgery and the first evidence of tumor relapse. Follow-up data collected until December 2017 were included in the analysis.

**TMA analysis and immunohistochemistry**

TMA (Outdo Biotech, Shanghai, China) was construct as previous described^1^. Immunohistochemical staining was performed using a primary antibody specific to BCL11B, as described previously^2^. Two pathologists independently scored the BCL11B immunostaining, which appeared mainly in the cell nuclei. The immunostaining proportion and intensity were used to determine the BCL11B expression in the tissue samples. The proportion of BCL11B staining was scored as follows: 0, <10% positive cells; 1, 10–40% positive cells; 2, 40–70% positive cells; 3, >70% positive cells. The intensity of BCL11B staining was scored as: 0, no staining; 1, weak staining; 2, intermediate staining; 3, strong staining. When the sum of the proportion and intensity scores was 8 or more, the expression status was defined as “high expression.”

**Cell lines and transfection**

Three human HCC lines (MHCC97L, HepG2, and Huh7) were purchased from the Chinese Academy of Sciences (Shanghai, China). All cancer cells were cultured in DMEM (Gibco BRL, Grand Island, NY) supplemented with 10% FBS (Gibco BRL, Grand Island, NY) at 37°C with 5% CO2. Transfection and lentivirus preparation were performed as previously described^2,3^. The plasmids HepG2-Mock, HepG2-sh1, HepG2-sh2, MHCC97L-Mock, MHCC97L-sh1, MHCC97L-sh2, Huh7-Mock, Huh7-OE were constructed by Shanghai Genechem Corporation (Shanghai, China). The plasmids shP73#1, and shP73#2 were constructed by Merdobio Corporation (Shanghai, China). SiE2F1 were constructed by Shanghai Gnenpharm Corporation (Shanghai, China). The target sequences are listed in Supplementary Table 3.

**RNA extraction, RT-PCR, and Western blot analysis**

RNA extraction, RT-PCR, and Western blot analysis were carried out using standard procedures descried elsewhere^4^. The primer sequences and antibodies used are shown in Supplementary Table 4 and Supplementary Table 5.

**Spheroid-formation assay**

Tumor cells (1,000 cells for each cell line) were seeded onto Ultra Low Cluster six-well plates (Corning) and cultured in DMEM/F12 medium supplemented with B-27 (1×) (Gibco BRL, Grand Island, NY), 20 ng/ml b-FGF, 20 ng/ml EGF and 10 ng/ml HGF (Peprotech).

**Cell proliferation and migration assays**

Cell proliferation was determined using colony-formation assays and CCK8 kits (Beyotime, Shanghai, China). For the colony-formation experiments, cells transfected with the appropriate plasmids were seeded onto six-well plates (1,000 cells per well) and cultured with complete DMEM for 12 days. The cells were then stained with Giemsa dye, and the colonies were counted. The CCK8 assay for cell proliferation was performed according to the manufacturer’s protocol. Absorbance was observed at 450 nm. Cell migration and invasion were detected using transwell chambers (BD, PharMingen, San Jose, CA) as previously described^5^. Additionally, cell migration was measured in a wound-healing assay^2^.

**Flow cytometry**

For analysis of apoptosis, control cells and experimental cells were seeded into 24-well plates and cultured for 48 h. Then, the cells were collected, resuspended, and treated with the Annexin V-FITC Apoptosis Detection Kit according to the manufacturer’s instructions (BD, PharMingen, San Jose, CA). Briefly, 5 μl FITC-labeled Annexin V and 5 μl propidium iodide (PI) solution were added to each sample, and the cells were incubated for 15 min at room temperature. For cell cycle analysis, cells were harvested and fixed in 70% ethanol overnight at 4°C. The cells were then washed three times, and 50 μg/ml PI, 100 μg/ml RNase A, and 0.2% Triton X-100 were added to each sample. The cells were then incubated for 30 min at room temperature in the dark and subsequently analyzed using an Aria II flow cytometer (BD, USA). For analysis of CD24+ and CK8+ cells, cell populations were stained with phycoerythrin-conjugated anti-human CD24 antibody and FITC-conjugated anti-human CK8 antibody, respectively. Cells treated with isotype-control rabbit immunoglobulin served as negative controls.

**Immunofluorescence**

For staining of CSC-related markers (CD24 and CK8)^6^ cells were fixed in 4% paraformaldehyde, blocked with BSA (5%), and incubated with CD24 or CK8 primary antibodies overnight at 4°C. The next day, the cells were stained with FITC-conjugated goat anti-rabbit secondary antibodies for 2 h at room temperature and then counterstained with DAPI (Sigma-Aldrich, USA).

**Chemoresistance assay**

Cells were exposed to sorafenib (5 μM) or doxorubicin (2 μM) for 24 h. The chemoresistance of the cells was determined by colony-formation assay (4,000 cells per well on six-well plates) and by apoptosis assay, as described in the preceding sections.

**Luciferase reporter assay**

A Cignal Finder Center 10-Pathway Reporter Array (QIAGEN, 336821) was used to investigate the mechanisms by which BCL11B suppresses CSC traits in HCC. E2F1 cDNAs were cloned in pcDNA3 (Invitrogen, Shanghai). Luciferase activity was measured using a luciferase reporter system. pGL2-P73 contained the promoter of P73 in pGL2-Basic (Promega, USA). Experimental protocols were conducted according to the previous study^7^. In order to test the ability of BCL11B to transactivate the P73 gene, Huh7 cells were transiently co-transfected with the pGL2-P73 plasmid and the BCL11B lentiviral vehicle. The silenced RNA control and silenced E2F1 RNA were transfected into Huh7 cells after 24h.

**Reference**

1. Ma X. l., Shen M. N., Hu B., Wang B. L., Yang W. J., Lv L. H., et al. CD73 promotes hepatocellular carcinoma progression and metastasis via activating PI3K/AKT signaling by inducing Rap1-mediated membrane localization of P110β and predicts poor prognosis. J HEMATOL ONCOL. 12，1 (2019). `

2. Gao Q., Zhao Y. J., Wang X. Y., Guo W. J., Gao S., Wei L., et al. Activating mutations in PTPN3 promote cholangiocarcinoma cell proliferation and migration and are associated with tumor recurrence in patients. GASTROENTEROLOGY. 146，1397-407 (2014).

3. Liu Y., Zhang J. B., Qin Y., Wang W., Wei L., Teng Y., et al. PROX1 promotes hepatocellular carcinoma metastasis by way of up-regulating hypoxia-inducible factor 1alpha expression and protein stability. HEPATOLOGY. 58, 692-705 (2013).

4. Marquardt J. U., Raggi C., Andersen J. B., Seo D., Avital I., Geller D., et al. Human hepatic cancer stem cells are characterized by common stemness traits and diverse oncogenic pathways. HEPATOLOGY. 54, 1031-42 (2011).

5. Akita H., Marquardt J. U., Durkin M. E., Kitade M., Seo D., Conner E. A., et al. MYC activates stem-like cell potential in hepatocarcinoma by a p53-dependent mechanism. CANCER RES. 74, 5903-13 (2014).

6. Ma X. l., Sun Y. F., Wang B. L., Shen M. N., Zhou Y., Chen J. W., et al. Sphere-forming culture enriches liver cancer stem cells and reveals Stearoyl-CoA desaturase 1 as a potential therapeutic target. BMC CANCER. 19, 760 (2019).

7. Flinterman M., Guelen L., Ezzati-Nik S., Killick R., Melino G., Tominaga K., et al. E1A activates transcription of p73 and Noxa to induce apoptosis. J BIOL CHEM. 280, 5945‐5959 (2005).
